# Insights Into the Role of Platelet-Derived Growth Factors: Implications for Parkinson’s Disease Pathogenesis and Treatment

**DOI:** 10.3389/fnagi.2022.890509

**Published:** 2022-07-01

**Authors:** Dan Li, Le-Tian Huang, Cheng-pu Zhang, Qiang Li, Jia-He Wang

**Affiliations:** ^1^Department of Family Medicine, Shengjing Hospital of China Medical University, Shenyang, China; ^2^Department of Oncology, Shengjing Hospital of China Medical University, Shenyang, China; ^3^Department of Laboratory Medicine, Shengjing Hospital of China Medical University, Shenyang, China

**Keywords:** platelet-derived growth factor, Parkinson’s disease, dopaminergic neurons, oxidative stress, calcium homeostasis

## Abstract

Parkinson’s disease (PD), the second most common neurodegenerative disease after Alzheimer’s disease, commonly occurs in the elderly population, causing a significant medical and economic burden to the aging society worldwide. At present, there are few effective methods that achieve satisfactory clinical results in the treatment of PD. Platelet-derived growth factors (PDGFs) and platelet-derived growth factor receptors (PDGFRs) are important neurotrophic factors that are expressed in various cell types. Their unique structures allow for specific binding that can effectively regulate vital functions in the nervous system. In this review, we summarized the possible mechanisms by which PDGFs/PDGFRs regulate the occurrence and development of PD by affecting oxidative stress, mitochondrial function, protein folding and aggregation, Ca^2+^ homeostasis, and cell neuroinflammation. These modes of action mainly depend on the type and distribution of PDGFs in different nerve cells. We also summarized the possible clinical applications and prospects for PDGF in the treatment of PD, especially in genetic treatment. Recent advances have shown that PDGFs have contradictory roles within the central nervous system (CNS). Although they exert neuroprotective effects through multiple pathways, they are also associated with the disruption of the blood–brain barrier (BBB). Our recommendations based on our findings include further investigation of the contradictory neurotrophic and neurotoxic effects of the PDGFs acting on the CNS.

## Introduction

Second only to Alzheimer’s disease (AD), Parkinson’s disease (PD) is a common neurodegenerative disease worldwide (Ascherio and Schwarzschild, 2016). The main risk factor in PD is age, and its prevalence is estimated to be almost 0.3% in the general population of industrialized countries. In people over the age of 60, the prevalence of PD is 1%, and, in people over the age of 80, the prevalence is 3%. Based on prospective studies, the incidence of PD is approximately 8–18 per 100,000 people per year, the median age of the onset is 60 years, and the progression time from diagnosis to death is almost 15 years (Nussbaum and Ellis, 2003; Tysnes and Storstein, 2017). On average, the age of the onset in men is almost 2.1 years earlier than that in women, and the incidence is almost 1.5–2 times that in women (Martinez-Martin et al., 2012). This might be explained by studies that have shown that estrogen may have a protective effect on striatal dopaminergic neuron cells, indicating that the phenotype of PD in women is generally milder and the incidence of dyskinesia is lower (Gillies et al., 2014). Recently published data have indicated that the rising risk of PD is associated with global aging trends (Seljeseth et al., 2000). It is also speculated that the increased risk of PD may be related to changes in smoking behavior in the late 20th century and increased traffic-related air pollution (Lee et al., 2016b; Savica et al., 2016). Yang et al. (2020a) found that approximately 1 million people in the United States were diagnosed with PD in 2017, resulting in an economic burden of $51.9 billion. As such, in addition to posing a significant threat to human health, PD represents a serious economic burden; therefore, more research is urgently needed to find treatments that can prevent and/or reverse the progression of PD.

**FIGURE 1:**
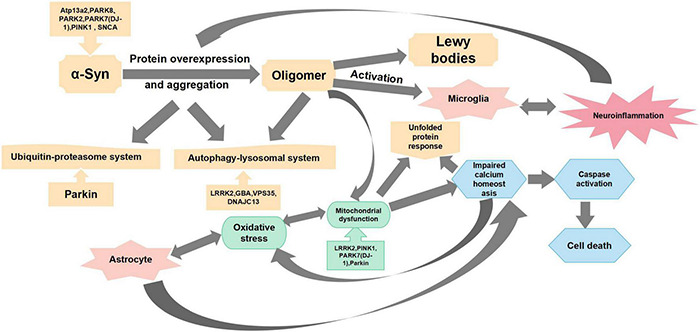
Main mechanisms of Parkinson’s disease pathogenesis. This figure summarizes the mechanisms of Parkinson’s disease pathogenesis from oxidative stress, mitochondrial dysfunction, protein overexpression and aggregation, neuroinflammation, and impaired calcium homeostasis.

Cell death and atrophy in specific regions of the brain contribute to the basic pathological features of different neurodegenerative diseases. The pathophysiological mechanism of PD is mainly manifested *via* the selectivity of dopamine neurons in the midbrain substantia nigra (SN)-striatal pathway and the accumulation of Lewy bodies in the remaining neurons (Obeso et al., 2000; Chi et al., 2018). Studies have shown that “Parkinsonism” occurs when approximately 50–60% of SN neurons are lost, and 80–85% of dopamine in the striatum is depleted (Maraganore et al., 2004). All major signs of PD are associated with motor dysfunction, which includes resting tremors, bradykinesia, rigidity, and postural disturbances. Non-motor dysfunction manifestations include dysphrenia (e.g., anxiety and depression) and autonomic dysfunctions (e.g., hypotension, constipation, paresthesia, spasticity, olfactory dysfunction, and seborrheic dermatitis). In addition, disease progression may lead to cognitive decline in patients (Wird Ef Eldt et al., 2011). Selective degeneration of nigrostriatal dopaminergic neurons and their fibers in PD is a gradual process. Therefore, successful protection, regeneration, and functional recovery of the nigrostriatal dopamine pathway may delay PD progression. The pathogenesis of PD is mainly manifested *via* the degeneration of dopaminergic neurons, including defects in mitochondrial function, oxidative stress, impaired Ca^2+^ homeostasis, protein misfolding and aggregation, and neuroinflammation. Additionally, changes in genetic factors and glial cell proliferation are also intimately associated with the occurrence of PD (Figure 1).

**FIGURE 2:**
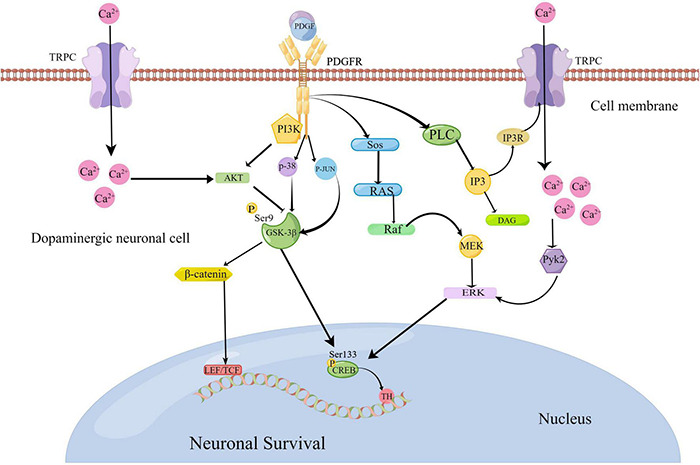
Platelet-derived growth factor (PDGF)-mediated pathological signaling mechanisms in Parkinson’s disease (www.figdraw.com). The binding of PDGF ligands and receptors can activate the PI3K/AKT/GSK-3β and MEK/ERK pathways to activate the transcription factor cycle response element binding protein (CREB) to promote the generation of tyrosine hydroxylase (TH). PDGFs can activate the P38 and JNK MAPK/GSK-3β/β-catenin signaling cascade. They can also activate the PLC/PI3R pathway to further activate the transient receptor potential canonical (TRPC) channel. As a result, Ca^2+^ elevation is triggered to suppress the GSK3β signal, which further leads to the accumulation of β-serial protein and gene expression related to cell survival. At the same time, elevated Ca^2+^ can activate the PYK2/ERK pathway, resulting in CREB activation.

In recent years, progress has been made in understanding the relationship between platelet-derived growth factors (PDGFs) and neurodegenerative diseases. PDGFs protect dopamine neurons, and this has been shown to play a role in PD. For example, Chen et al. (2021b) showed that the signaling pathways PI3K/Akt/GSK-3β and MEK/extracellular signal-regulated kinase (ERK) are involved in the process of MPP + toxicity after PDGF-BB treatment, which contribute to the phosphorylation and nuclear translocation of downstream effector cycle response element binding protein (CREB) (Chen et al., 2021b). Zheng et al. (2010) further confirmed that the nerve protective effect of PDGF-AA in this pathway is slightly weaker than that of PDGF-BB (Zheng et al., 2010). Recently, Chen et al. (2021a) have also confirmed that PDGF-BB promotes the generation of tyrosine hydroxylase (TH) by activating the transcription factor CREB, which transfers to the nucleus and is combined with the starting sub-region of TH genes in dopamine neurons (Chen et al., 2021a). Peng et al. (2012) verified that PDGF-CC can activate the PLC/PI3R pathway, thereby activating the transient receptor potential canonical (TRPC) channel and triggering Ca^2+^ elevation. This increase in Ca^2+^ inhibits the GSK3β signal in the PI3K/AKT pathway induced by PDGF, which further leads to β-catenin accumulation in the cytoplasm and, subsequently, induces gene expression related to cell survival (Peng et al., 2012). Chao et al. (2014) confirmed that PDGF-BB activates the P38 and Jun N-terminal kinases (JNK) mitogen-activated protein kinase (MAPK)/GSK-3β/β-catenin signaling cascade, thereby promoting the spread of neural progenitor cells (NPCs)(Chao et al., 2014). Furthermore, Yao et al. (2009) reported that PDGF-mediated PDGF-β receptors activate the PLC/IP3R pathway, which activates TRPC channels, increases Ca^2+^, and activates the Pyk2/ERK pathway that leads to CREB activation, resulting in a protective effect on rat primary neurons (Yao et al., 2009). These signaling pathways linked to PDGFs are summarized in Figure 2.

In addition to the role of PDGFs in the signaling pathways associated with PD, we also summarized the effects of PDGFs on PD progression in this review. We further illustrated the possible applications and prospects of PDGFs for PD, with a focus on targeted gene therapy.

## Platelet-Derived Growth Factors

As a type of neurotrophic factors (NTFs), PDGFs are important mitogen and chemotactic agents. PDGFs can be expressed in mesenchymal cells, osteoblasts, and vascular smooth muscle cells (VSMCs) (Carl-Henrik and Bengt, 1999). NFTs are a class of proteins with a molecular weight of 10–35 kDa that play active roles in neuronal development, differentiation, survival, and plasticity (Huttunen and Saarma, 2019). Crucially, various NFTs have been shown to restore the dopaminergic nigrostriatal pathway, which is impaired in patients with PD.

Since Balk (1971) observed that normal chick embryo fibroblasts do not grow rapidly in low-calcium and platelet-free plasma while normal fibroblasts grow well after replacing plasma with serum, considerable research has followed on the growth-stimulating factors present in serum. It was found that platelets are the source of growth-stimulating activity, and their extracts can promote the growth and proliferation of fibroblasts, smooth muscle cells, and glial cells (Balk, 1971; Kohler and Lipton, 1974; Westermark and Wasteson, 1976; Aso et al., 1980). On this basis, PDGFs, also known as glioma-derived growth factors and osteosarcoma-derived growth factors, were successfully isolated and purified.

### Classification and Structure of Platelet-Derived Growth Factors

**FIGURE 3:**
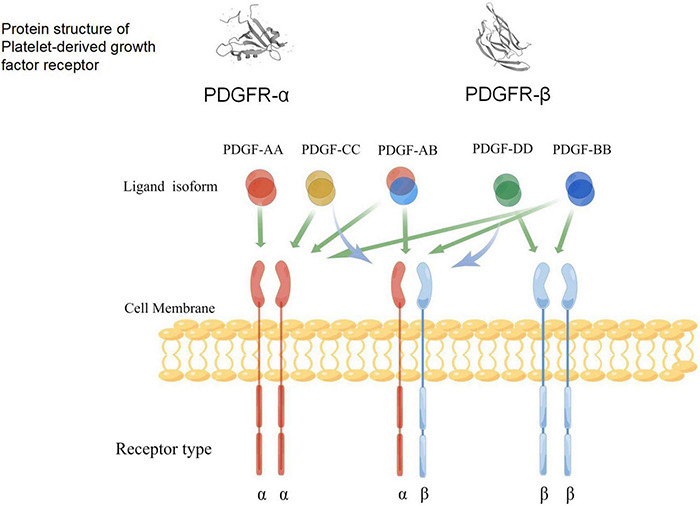
A schematic diagram of the specific binding of platelet-derived growth factor (PDGF) ligand to the receptors, and the structure of its receptors (www.figdraw.com). Both PDGFR-α and PDGFR-β include an extracellular domain that combines with ligands, a single cross-membrane domain of extracellular signals to the intracellular regions, internal intracellular segments, kinase domain, and C-end acidic tail. Green arrows indicate the ability of different PDGF isoforms to bind and activate the dimeric receptor complex; blue arrows indicate the ability to activate receptor heterodimers.

Platelet-derived growth factors are a family of cystine-knot-type growth factors composed of five functional subunits. Their structure is made up of highly homologous polypeptide chains (A, B, C, and D) that are formed by disulfide bonds (Kazlauskas, 2017; Huttunen and Saarma, 2019). These four types of polypeptide chains are encoded by four genes, among which PDGF-B was first identified *via* amino acid sequencing and presents a high homology with the simian sarcoma virus oncogene (Doolittle et al., 1983; Waterfield et al., 1983). The cDNA of PDGF-A was obtained by cloning, and its location was identified on Chromosome 7 (Betsholtz et al., 1986). The PDGF protein was discovered using biochemical methods; it is composed of PDGF-A and PDGF-B dimer proteins, as well as PDGF-AB heterodimers. In the early 2000s, genetic and biochemical methods were used to identify new ligands activated by PDGF-C and PDGF-D (Li et al., 2000; Bergsten et al., 2001; LaRochelle et al., 2001). As a receptor for PDGF, platelet-derived growth factor receptor (PDGFR) was also discovered in humans through cross-linking studies (Klinghoffer et al., 2002). Studies have also shown that PDGFR-α and PDGFR-β share common promoter proteins with c-Kit, c-Fms, and FLT (Kazlauskas and Cooper, 1989; Andrae et al., 2008). The five dimer isomers of PDGF have different affinities for the two PDGF tyrosine kinase receptors (TKRs) (Fredriksson et al., 2004; Janneth, 2015; Huttunen and Saarma, 2019); therefore, there are many possible interactions between PDGFs and PDGFRs (Figure 3). The phosphorylation of tyrosine residues in intracellular domains can be promoted by the polymerization of the associated subunit caused by the binding between the PDGF ligand and the receptor (Andrae et al., 2008; Janneth, 2015). Studies have shown that PDGFR-α and PDGFR-β share a common structure—five extracellular immunoglobulin (IG)-type domains and one intracellular tyrosine kinase domain (Figure 3). Various signaling pathways and mediators are activated by receptor phosphorylation, including MAPK, phosphoinositide 3-kinase (PI3K), the Wnt pathway, and phospholipase C (PLC). It has also been confirmed that the A-, B-, and C-polypeptide chains of PDGF can bind to PDGFR-α with high affinity, which means that PDGFR-α can be activated by homodimers PDGF-AA, PDGF-BB, and PDGF-CC, and the heterodimer PDGF-AB. The B- and D-polypeptide chains of PDGF can bind to PDGFR-β with high affinity, which means that PDGFR-β can only be activated by PDGF-BB and PDGF-DD (Carl-Henrik and Bengt, 1999). PDGFR-αβ heterodimers can be induced by PDGF-BB homodimers or PDGF-AB heterodimers. These interaction patterns suggest that PDGFR-α is more promiscuous than PDGFR-β, whereas PDGF-B is more promiscuous compared to other PDGFs. Smaller, less conformationally specific residues at the ligand–receptor interface account for the promiscuous nature of PDGFR-α, with fewer aromatic and hydrophobic residues than PDGFR-β, which has abundant aromatic residues on its ligand-binding surface. The presence of a large number of long-chain hydrophilic residues at the edge of the receptor-binding surface of PDGF-B explains its promiscuity (Lo Conte et al., 1999). PDGF-C is the only member of the PDGF family that has a propeptide-independent recombinant expression of growth factor domains (Shim et al., 2010), the receptor-binding surface, which may be more hydrophilic compared to that of other PDGFs. Thus, PDGF-C can adapt more easily to the ligand-binding surface of PDGFR-α. Finally, compared with other PDGFs, PDGF-CC is most similar to vascular endothelial growth factors (VEGFs), which implies that its underlying function is different from other PDGF members (Reigstad et al., 2005).

### Distribution and Biological Functions of Platelet-Derived Growth Factors

Platelet-derived growth factors can be synthesized in various cells, including brain cells, such as neuronal progenitor cells, neuronal cells, astrocytes, and oligodendrocytes, and mainly function by either autocrine or paracrine stimulation. In the processes of wound repair, angiogenesis, and atherosclerosis, cell cycle and gene expression patterns can be regulated by PDGFs. The normal blood vessel wall expresses low levels of PDGF under physiological conditions, whereas, when the intima is damaged, a local increase in PDGF levels predicts adverse remodeling after vascular injury (Andrae et al., 2008). PDGFs can bind to PDGF receptors (PDGFR-α and PDGFR-β) through receptor tyrosine kinase (RTK) activity, which can bind to ligands and phosphorylate tyrosine residues of target proteins, stimulate receptor dimerization, and initiate intracellular signal transduction during biological functions (Chen et al., 2013). PDGF-A transcripts are expressed in the brain during late embryonic development of most neurons, which precedes the differentiation of most glial cells. While proteins containing PDGF-B chains have been found to localize in neurons throughout the CNS of adult non-human primates, such as *Macaca nemestrina* (Hart et al., 1989), its positive immunohistochemical staining reaction is limited to neuronal perinuclear regions and dendrites. The strength of this response varies with the location of the neuron; blood vessels stain weakly, while glial cells are not stained (Sasahara et al., 1991). PDGF-C and PDGF-A are co-expressed in heart, brain, liver, kidney, and testes, and PDGF-C is widely synthesized in various tissue cells of mouse embryos, including somites, craniofacial mesenchymal cells, cardiomyocytes, arterial smooth muscle cells, cartilage, mast cartilage cells, and the CNS. The expression of PDGF-C is also related to the formation of glandular ducts during embryonic development (Aase et al., 2002), and, in the adult nervous system, it is expressed in cerebellar neurons, anterior olfactory nucleus, pontine nuclei, cochlear neuronal cells, astrocytes, microglia, oligodendrocytes, and oligodendrocyte precursor cells (OPCs) (Tian et al., 2021). PDGF-D is widely synthetized in normal human tissues, and exhibits a high degree of expression in adrenal tissue; moderate expression in pancreas, adipose, heart, stomach, bladder, trachea, breast, ovary, and testis tissues; some degree of expression in brain, pituitary, liver, lung tissues; and low or no expression in small intestine, colon, skeletal muscle, thyroid, salivary gland, or thymus tissues (LaRochelle et al., 2001). PDGF-D can also be synthetized in VSMCs, endothelial cells, kidney epithelial cells, and fibroblasts (Borkham-Kamphorst et al., 2015).

Platelet-derived growth factor receptors, including PDGFR-α and PDGFR-β, are synthetized by various neuronal cell types during nervous system development, such as dopaminergic neurons in the SN, cortical neurons, striatal neurons, neurospheres, retinal ganglion cells, and neuronal cells in the inward and outer nuclear layers of the retina (Oumesmar et al., 1997).

PDGFR-α and PDGFR-β are class III RTKs (Lemmon and Schlessinger, 2010), although their expression patterns and physiological roles differ. For example, PDGFR-α signaling pathways regulate the development and formation of gastrula, neuroprotective tissues, and various organs, whereas PDGFR-β receptor expression is required for embryonic neural crest development, astrocyte development and differentiation, and dendritic spine morphogenesis and plasticity (Svitkina et al., 2010; Funa and Sasahara, 2014). In addition, the PDGFR-β signaling pathway plays an important role in the early stages of hematopoiesis and angiogenesis. Furthermore, a wide variety of mesenchymal cells are influenced in PDGFR-α-null embryos, while the deletion of embryonic PDGFR-β results in a lack of smooth muscle cells, particularly VSMCs and pericytes (Wu et al., 2008).

PDGF-BB is expressed in platelets, megakaryocytes, fibroblasts, smooth muscle cells, neurons, oligodendrocytes, and astrocytes (Krupinski et al., 1997; Yu et al., 2001, 2003; Board and Jayson, 2005; Kang, 2007; Trojanowska, 2008). As an important mitogenic factor, PDGF-BB is important for the induction of embryonic and vascular development, wound healing *in vivo*, chemotaxis regulation, and cell transformation *in vitro* (Yu et al., 2003; Andrae et al., 2008). PDGF-CC is widely expressed in different types of neuronal tissues, including the brain, eyes, and spinal cord (Hamada et al., 2000; Hao et al., 2000; Aase et al., 2002; Lei et al., 2007). Ding et al. (2004) also showed that a lack of PDGF-CC in mice leads to postnatal developmental defects and death, indicating that PDGF-CC is required for embryonic development (Ding et al., 2004).

## Platelet-Derived Growth Factor Roles in Parkinson’s Disease

**FIGURE 4:**
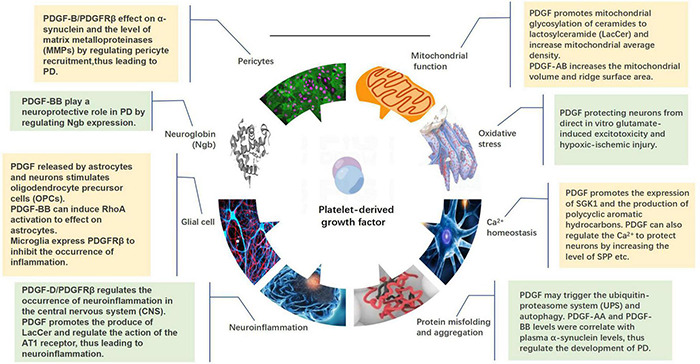
Mechanisms by which platelet-derived growth factors (PDGFs) act on the development of Parkinson’s disease, including mitochondrial function, oxidative stress, calcium homeostasis, protein misfolding and aggregation, neuroinflammation, glia, neuroglobin, and pericytes.

Platelet-derived growth factor regulates the functional activities of neurons by regenerating, stabilizing, and stimulating the synapses of neuronal axons, thereby regulating the synthesis and release of neurotransmitters and affecting the expression of related transport proteins. The use of different PDGF isoforms in different research models demonstrated their potential in the protection and regeneration of specific neural cells (Iihara et al., 1997; Krupinski et al., 1997; Tang et al., 2010; Peng et al., 2012; Vasefi et al., 2012; Paul et al., 2015). This protective effect may be achieved by regulating mitochondrial function, oxidative stress, Ca^2+^ homeostasis, protein misfolding and aggregation, and neuroinflammation. PDGFs can also act on glial cells, neuroglobins (Ngb), and pericytes to affect the progression of PD (Figure 4).

### Platelet-Derived Growth Factors Regulate Mitochondrial Function

Mitochondria balance cell death and survival, which is particularly important for maintaining aerobic balance in neurons in the brain, which consume 2% of the body’s total oxygen. The energy required to maintain the ionic gradient across nerve elementary membranes drives this oxygen demand, which also generates action potential. This demand for energy reflects the importance of mitochondrial function in neuronal cells, and, therefore, mitochondrial dysfunction, mitochondrial DNA mutations, mitochondria-related DNA gene mutations, and the presence of mitochondria-related mutant proteins may be associated with PD. Mitochondria involved in the generation of adenosine triphosphate (ATP) through oxidative phosphorylation participate in the regulation of intracellular calcium ion levels and control membrane excitation and neurotransmission, and, thereby, regulate cell energy metabolism. Thus, mitochondrial damage results in blocked ATP production and elevated reactive oxygen species (ROS), which are one-electron reduction products of a class of oxygen *in vivo* that can modulate intracellular calcium levels and damage dopaminergic neurons (Rose et al., 2017; Norat et al., 2020). Furthermore, PD is associated with mitochondrial deletion and apoptosis activation (Simpkins et al., 2010). Importantly, mitochondria have different physiological properties in different tissues. For example, mitochondria isolated from the liver are unable to produce free radicals, which, in turn, causes mitochondria in the brain to produce high amounts of oxygen- and carbon-centered free radicals (Dykens, 2007). Mitochondrial damage also impedes the transport of long-distance organelles, rendering neurons with long axons and/or dendrites more vulnerable (Chang and Reynolds, 2006). In addition to proximal mitochondrial damage, the selective susceptibility of different mitochondrial defects in different brain regions may also be related to other factors, such as dopamine- and iron-rich SN, which can jointly exacerbate the oxidative stress caused by catecholamine autoxidation, as well as mitochondrial dysfunction (Hermida-Ameijeiras et al., 2004).

Ceramide can inhibit mitochondrial respiratory chain activity and regulate the permeability of the mitochondrial inner membrane, although its production is not sufficient to induce apoptosis and necrosis in sphingomyelinase-deficient mutant cells or mitochondria (Martin et al., 2006). PDGFs promote mitochondrial glycosylation of ceramides to lactosylceramide (LacCer), which has been shown to potentially affect the function of mitochondria in nerve cells in various ways (Chatterjee et al., 2021). Novgorodov et al. (2016) also found that ceramide synthase activity is not proportional to ceramide levels, while LacCer levels are elevated. This confirmed the dominance of the glycosylation pathway in diabetic mice, showing that the synthesis of LacCer reduces ceramide levels. LacCer, one of the effectors of mitochondrial function, has also been shown to effectively inhibit State 3 respiration and reduce calcium retention capacity (CRC) in baseline mitochondria, resulting in mitochondrial dysfunction (Novgorodov et al., 2016).

The treatment of injured fibroblasts with PDGF-AB increases mitochondrial volume and the ridge surface area, reflecting its ability to induce ultrastructural changes associated with increased energy demand (Gosslau et al., 2001). Indeed, Gosslau et al. (2001) found that application of PDGF to NRK-49F fibroblasts led to a 57% increase in mitochondrial bulk density and a 65% increase in the cristae surface area compared to controls. At the same time, while the density of mitochondria in PDGF-treated cells decreased by 23%, their mean volume increased. These observations demonstrate the effect of PDGF on the mitochondria of nerve cells, which requires further research.

In conclusion, the existing research on the effects of the PDGF family on cell survival by affecting mitochondrial function remains limited and, in some cases, contradictory. As such, there are multiple promising avenues for future research on PDGFs.

### Platelet-Derived Growth Factors Regulate Oxidative Stress

Regulate oxidative stress (ROS) generated by cells under oxidative stress in patients with PD can target and attack mitochondria. The long unmyelinated axons of the SN dopaminergic neurons increase energy consumption; this energy state leads to the damage and death of dopaminergic neurons, which leads to compensation by the residual neurons by accelerating the synthesis, metabolism, and renewal rate of dopamine. This further leads to the generation of more oxidative free radicals and further increases oxidative stress, thereby promoting PD progression (Jenner, 2003; Dias et al., 2013; Pissadaki and Bolam, 2013). High levels of ROS, nitric oxide (NO), interleukin 1β (IL-1β), interleukin 6 (IL-6), and tumor necrosis factor alpha (TNF-α) are neurotoxic, and further activation of the apoptotic pathway *via* the action of cytochrome C and caspase 3 affects the mitochondrial energy production in dopaminergic neurons (Menza et al., 2010). The cumulative effect of these processes can cause degenerative changes in the associated neurons (Wachter et al., 2010).

Many studies have demonstrated that PDGFs exert neuroprotective effects by protecting neurons and brain cells from direct *in vitro* glutamate-derived excitotoxicity and hypoxic-ischemic (H-I) injury. Neuronal excitotoxic death can result from overstimulation *via* elevated levels of extracellular glutamate acting on N-methyl-D-aspartate (NMDA) and alpha-amino-3-hydroxy-5-methyl-4-isoxazolepropionic acid (AMPA) receptors. The PDGF-B chain protects neurons by inhibiting NMDA-induced currents and transporting glutamate transporters to the cell membrane. It was found that the balance between N-methyl-D-aspartate receptor (NMDAR) and PDGF-B expression partly contributes to an individual’s susceptibility to brain injury. Furthermore, enhancement of PDGF-B/receptor signaling may protect neonatal brains from H-I injury. Tseng and Dichter (2005) found that PDGF-BB preincubation weakens neuronal death caused by transient exposure to glutamate or NMDA. This protection is concentration- and time-dependent, and excitatory neurons were selectively protected (Egawa-Tsuzuki et al., 2004; Tseng and Dichter, 2005; Ishii et al., 2010). Other signaling mechanisms mediated by signal transducers and activators of transcription (STATs)and activated by PDGF ligands are important for cell proliferation, differentiation, survival, and transformation (Kang, 2007). For example, in vascular muscle cells, the activation of STAT 1, 3, and 6 by PDGF ligands occurs during airway remodeling in asthma *via* the activation of transmembrane NOX enzymes (nicotinamide adenine dihydrogen phosphate (NADPH) oxidase/dioxidase), leading to the production of hydrogen peroxide (H_2_O_2_) independent from ROS produced by the mitochondria (Simon et al., 2002). H_2_O_2_ acts as a secondary messenger in the STAT-activated signaling pathway and is important for the regulation of downstream phosphatase proteins. Therefore, H_2_O_2_ produced by PDGF ligands may have beneficial effects (Thannickal and Fanburg, 2000). This possible cellular protective effect of PDGF has not yet been verified in any PD model. It was shown that the transient increase in ROS levels following PDGF administration is likely attributable to the induction of membrane NOX enzymatic complexes. PDGF-promoted LacCer synthesis also activates NADPH oxidase to produce ROS, leading to a high oxidative stress environment that stimulates a range of signaling molecules and pathways, resulting in inflammation and atherosclerosis (Pagano et al., 1997). Whether different PDGF isoforms are involved in ROS generation in the nervous system has not yet been determined nor validated in PD models.

Microarray analysis of human umbilical vein endothelial cells (HUVECs) showed that oxidized low-density lipoprotein (oxLDL) induces high expression of PDGF-A and PDGFR-α after 6 h of pretreatment (Virgili et al., 2003). Bovine aorta endothelial cells (BAECs) driving firefly luciferase activity with a 643-bp PDGF-A promoter and exposed to oxLDL and native LDL (40 μg/ml) also showed increased PDGF-A promoter activity compared to vector-transformed cells. Increased expression of PDGF-A, PDGFR-α, and PDGFR-β has also been observed in response to oxLDL in VSMCs (Stiko-Rahm et al., 1992). PDGF-BB preconditioning for 24 h was shown to have significant protective effects against H_2_O_2_, glucose deprivation, and excitotoxic injury in cultured neurons, as demonstrated in most neuronal models (Cheng and Mattson, 1995; Tseng and Dichter, 2005; Zheng et al., 2010). Cabezas et al. (2015; 2016; 2018) found that pretreatment with PDGF-BB (200 ng/ml) can maintain mitochondrial membrane potential (Δψm) by decreasing the generation of superoxide and peroxide radicals. This, in turn, protects human astrocyte T98G cells from rotenone (50 μM), maintains mitochondrial ultrastructure, and boosts the activation of the PI3K/AKT signaling pathway (Cabezas et al., 2015, 2016, 2018). Northern blot analysis of BAECs exposed to H_2_O_2_ (25 and 50 μM) for 30 min also demonstrated a significant increase in PDGF-B mRNA expression (Montisano et al., 1992). An early increase in PDGF-B mRNA levels was also observed by expression analysis of (total) RNA in rat lung cells extracted after oxidative injury (Fabisiak et al., 1989). Furthermore, oxLDL enhances PDGF-B expression in HUVECs in a dose-dependent fashion (Zhao and Xu, 2000). In contrast, in BAECs and human monocyte-originated macrophages, oxLDL reduces PDGF-B expression (Van Heek et al., 1998).

Based on this existing research, the protective effects of PDGF for cells under oxidative stress have been widely confirmed. However, the particular responses of different subtypes and in certain cells still require further research.

### Platelet-Derived Growth Factors Regulate Ca^2+^ Homeostasis

Previous works have confirmed that dopaminergic neuron activity is affected by an imbalance in Ca^2+^ homeostasis and the modulation of Ca^2+^ carriers (Cieri et al., 2017). Changes in mitochondrial dynamics are also associated with the formation of contact sites in the endoplasmic reticulum. Effective endoplasmic reticulum–mitochondrial communication is required for the maintenance of mitochondrial bioenergetics, Ca^2+^ homeostasis, and cell survival (Paillusson et al., 2016). Thus, disruption of Ca^2+^ signaling molecules and other components may trigger excess Ca^2+^ influx, leading to the degeneration of dopaminergic neurons. Neuroinflammation can also lead to the abnormal expression and aggregation of α-synuclein (Kilpatrick, 2016). Mutual aggravation of α-synuclein and mitochondrial dysfunction, as well as mutual promotion of mitochondrial dysfunction and oxidative stress, further leads to impaired Ca^2+^ homeostasis, creating positive feedback. Such damaging effects are, therefore, continuously enhanced until caspases are activated, thereby inducing cell death (Tran et al., 2020). It has been reported that PDGF can participate in the production of polycyclic aromatic hydrocarbons, and, upon binding to its specific RTK, PLC is activated through a G protein-dependent or independent process. This results in an increase in diacylglycerol (DAG) and inositol triphosphate (IP3) levels, which results in the activation of PLC and the mobilization of intracellular Ca^2+^ (Clark et al., 1995; Yamamura et al., 2021). Cell proliferation, differentiation, and migration are subsequently induced. Serum and glucocorticoid-inducible kinase 1 (SGK1) expression can be stimulated by PDGF in megakaryocytes and circulating platelets. In megakaryocytes, SGK1 activates NF-κB, which causes the expression of calcium release-activated calcium channel 1 (Orai1). Orai1 is a Ca^2+^ channel protein that promotes Ca^2+^ entry, termed store-operated calcium entry (SOCE). SOCE and several Ca^2+^-sensitive platelet functions can be enhanced by SGK1, including degranulation, activation of integrin αIIbβ3, phosphatidylserine exposure, aggregation, and thrombosis (Lang et al., 2015). The level of sphingosine 1-phosphate (SPP), a phosphorylated derivative of sphingosine that acts as an intracellular secondary messenger, can rapidly and transiently increase under the action of PDGF to release Ca^2+^ from internal sources. This occurs independently of inositol trisphosphate receptor (InsP3R), and SPP may link sphingolipid signaling to cellular Ras-mediated signaling by increasing phosphatidic acid levels (Spiegel et al., 1994).

Numerous studies have indicated that, under normal and pathological conditions, intracellular calcium concentration can modulate PDGF-BB signaling (Ridefelt et al., 1995; Pinzani, 2002; Peng et al., 2012; Paul et al., 2015). In this regard, PLC can be activated by PDGF-BB, leading to the formation of IP3 and DAG, which increase the mobilization of Ca^2+^ from the intracellular compartment and lead to the activation of protein kinase C (Powis et al., 1990; Ridefelt et al., 1995). PDGFR-β activates calcium channels, suggesting that PDGF-BB promotes calcium influx and mitosis (Hammerman et al., 1993; Johnson et al., 1993; Prncpadscussan, 1993; Ling et al., 1995). These findings suggest that PDGF-BB has important neuroprotective properties. Indeed, the signaling mechanism of PDGF-BB involved in neuronal protection also includes the activation of anti-apoptotic and survival pathways, such as MAPK, PI3K-AKT, c-JNK, and NF-κB pathways (Romashkova and Makarov, 1999; Wang et al., 2010; Zheng et al., 2010). Calcium influx into cells modulates the activation of these signaling pathways, further affecting the phosphorylation of glycogen synthase kinase 3β (GSK3β) and β-catenin (Zhu et al., 2009; Peng et al., 2012). During cell growth, differentiation, apoptosis, and stress response, PDGF-BB can activate NF-κB by regulating the PI3K/AKT pathway while reducing the production of matrix metalloproteinase 9 (MMP-9) and VEGF (Romashkova and Makarov, 1999; Wang et al., 2010). In other words, the inactivation of GSK3β can be caused by the activation of PI3K/AKT. Furthermore, the inactive form of GSK3β prevents the degradation of β-catenin, thereby promoting its accumulation in the cytoplasm and translocation to the nucleus. This contributes to the activation of genes involved in cell survival (Peng et al., 2012). Additionally, β-catenin is involved in mitochondrial homeostasis, regulation of ATP production, and lipid oxidation (Lehwald et al., 2012; Arrázola et al., 2015). PI3K/AKT also negatively regulates the transcription factor forkhead (FOXO), which contributes to cell survival, oxidative stress regulation, and mitochondrial membrane polarization (Burgering and Medema, 2003; Kato et al., 2006). The importance of the PI3K/AKT pathway in these cellular metabolic processes is self-evident, reflecting the potential value of PDGF-BB.

### Platelet-Derived Growth Factors Regulate Protein Misfolding and Aggregation

Insoluble α-synuclein fibers are made of proteins composed of oligomers formed from soluble α-synuclein monomers, and, as these proteins aggregate, they form Lewy bodies (Melki, 2015). In the substantia nigra pars compacta (SNpc), the generation of Lewy bodies is one of the main pathological characteristics of primary PD. Oligomers formed by α-synuclein monomers can induce neuroinflammation by activating microglia, thereby promoting PD. The main pathological features of PD have been replicated in an α-synuclein mouse model (Hasegawa et al., 2017), and oxidation, nitration, the ubiquitin-proteasome system, and the lysosomal autophagy system were all found to be closely related to the degradation of α-synuclein (Wong and Krainc, 2017). The ubiquitin-proteasome system can degrade short-lived soluble proteins by regulating the gene encoding of the protein parkin (PRKN). The lysosomal autophagy system mainly degrades long-lived macromolecular proteins and affects the occurrence and development of PD by regulating the genes encoding the proteins leucine-rich repeat kinase 2 (LRRK2), β-glucocerebrosidase (GBA), vacuolar protein sorting-associated protein 35 (VPS35), and DNAJC13 (Maraganore et al., 2004). The unfolded protein response (UPR) is also involved in PD (Hashida et al., 2012). Elevated levels of ROS and calcium homeostasis imbalance during episodes of PD lead to changes in proteins containing α-synuclein, which stimulate the activation of UPR-regulated proteins, such as PERK kinase, inositol 1α-dependent enzyme (Ire1α), transcription factor 6α (ATF6α), and GRP78/Bip (Wang et al., 2009; Gorbatyuk et al., 2012; Sen, 2015). UPR is a pro-survival response as it reduces protein biosynthesis and enhances the degradation function of the endoplasmic reticulum, thereby reducing the endoplasmic reticulum burden and maintaining intracellular homeostasis (Walter and Ron, 2011). For the first time, Ishimura et al. (2014) demonstrated that PDGF-BB can induce UPR activation in the proliferation of coronary artery smooth muscle cells (CASMC) (Ishimura et al., 2014). Dihazi et al. (2013) also demonstrated that PDGF-stimulated renal fibroblasts or tubular cell lines generate ER stress and activate the UPR. This means that rapidly growing fibroblasts stimulated by cytokines can induce ER stress, leading to the protective UPR and increased expression of folding partners as a protective response (Dihazi et al., 2013).

PDGF-AA and PDGF-BB levels are associated with plasma α-synuclein levels in patients with PD (*p* < 0.0003), indicating their potential as PD biomarkers. Lue et al. (2016) biochemically validated that levels of thymus, activation-regulated chemokine (TARC), and PDGF-AA are significantly different between the patients with PD with and without dementia (Lue et al., 2016). Furthermore, under the regulation of PDGF promoter, transgenic mice overexpressing human α-Synuclein were generated by Masliah et al. (2000); 12 months later, these mice were found to have motion defects and a loss of dopamine (Masliah et al., 2000). Subsequently, Rockenstein et al. (2002) verified that, in the neocortex and limbic system, the PDGF promoter promotes the expression of human α-synuclein (Rockenstein et al., 2002). Şengül et al. (2021) further verified that overexpression of α-synuclein upregulates the secretion levels of PDGF-AA and PDGF-BB; while the expression of PDGFR-B in cells was shown to increase, PDGFR-B was predicted to interact with α-synuclein based on the Fp-Class PPI prediction tool. Based on Reactome pathway analysis, these authors found that PDGF may trigger the ubiquitin-proteasome system (UPS) and autophagy *via* the intracellular PI3K/Akt or MAPK pathway (Şengül et al., 2021). These studies demonstrate that PDGF may regulate the degradation of α-synuclein through UPR and autophagy.

### Platelet-Derived Growth Factors Regulate Neuroinflammation

The occurrence of PD caused by neuroinflammation is closely related to a variety of genes, including LRRK2. Studies have found that neuroinflammation can activate innate and adaptive immunity in PD by promoting the misfolding and aggregation of α-synuclein (Gao et al., 2008). Inflammatory responses in the olfactory system and gut tissue can also trigger high levels of α-synuclein misfolding, allowing α-synuclein aggregates to escape normal degradation mechanisms (Tomé et al., 2013). Sampson et al. (2016) demonstrated that inflammatory responses in the gut microbiota can promote microglial activation as well as α-synuclein pathological states and motor deficit states (Sampson et al., 2016).

The pathological process of inflammation is related to leukocyte extravasation and migration and involves cell-derived mediators (e.g., cytokines and adhesion molecules) (McCurley et al., 2013). Accumulating evidence suggests that neuroinflammation may play an important role in the pathological changes in patients with PD. PDGF-D can selectively agonize PDGFR-β isoforms, while, on the other hand, PDGFR-β affects macrophage activation, cell infiltration, and cell migration in CNS inflammation (Yang et al., 2016). Transactivation of PDGFR can be induced through the action of the AT1 receptor in VSMCs and tissues, which may support cell growth and migration (Heeneman et al., 2000; Schellings et al., 2006; Suzuki and Eguchi, 2006). PDGFR transactivation also mediates angiotensin II (Ang II)-induced ERK activation in mesangial cells. Ang II, however, depends on the AT1 receptor and acts through RTKs (PDGFR and endothelial growth factor receptor, EGFR) and non-RTKs [proto-oncogene tyrosine-protein kinase (Src), non-receptor protein-tyrosine kinase (Pyk2), and JAK/STAT]. AT1R-mediated activation of NADPH oxidase leads to the production of ROS, thereby promoting neuroinflammation. Simultaneously, these signaling cascades lead to the development and progression of glutamate excitotoxicity, apoptosis, cerebral infarction, astrocyte proliferation, nociception, neuroinflammation, and other neurological disease processes (Mondorf et al., 2000). LacCer synthase/LacCer has also been shown to play a role in cell proliferation, inflammation, and cancer (Asada et al., 1997). Using primary rat astrocytes, Pannu et al. (2004) demonstrated the potential role of LacCer synthase/LacCer in TNF-α-induced inflammation (Pannu et al., 2004). Astrocyte proliferation and LacCer synthase activity increase after stimulation with TNF-α; this was alleviated using D-threo-1-phenyl-2-decanoylamino-3-morpholino-1-propanol (D-PDMP), along with a 20-mer antisense oligonucleotide in rats. This led to reduced PI3K, Ras, and ERK1/2 expression, and the inhibition of astrocyte proliferation. LacCer also activates inducible nitric oxide synthase (iNOS), thereby promoting NO generation. NO is a neuronal messenger that becomes toxic at high concentrations and exacerbates the progression of several neurodegenerative diseases (Won et al., 2007).

### Other Mechanisms by Which Platelet-Derived Growth Factors Regulate Parkinson’s Disease Progression

#### Platelet-Derived Growth Factors Regulate Glial Cell Changes

Dopaminergic neurons play a major role in the progression of PD; however, there are reports that glial cells are also involved in its inflammatory and degenerative processes (McGeer and McGeer, 2008; Mena and Garcia de Yebenes, 2008; Ahmed et al., 2013; More et al., 2013). As the major cell type in the mammalian brain, astrocytes constitute glial cells along with oligodendrocytes and microglia (Chen and Swanson, 2003), and are part of a syncytial network, including pericytes, endothelial cells, and neurons (Bushong et al., 2002). Astrocytes play a significant role in the development and maintenance of the BBB, promoting neurovascular coupling, releasing chemokines and glial transmitters to recruit cells, regulating calcium release, and transporting glutamate. Astrocytes can send signals through the glutamate aspartate transporter and the excitatory amino acid transporter, thereby maintaining brain metabolism, regulating brain pH and specific transporter uptake of γ-aminobutyric acid, as well as producing antioxidant enzymes (Volterra and Meldolesi, 2005; Hamby and Sofroniew, 2010; Parpura et al., 2011). During brain injury (e.g., oxidative stress), these processes are affected to varying degrees, and their effects on neuronal cells can result in pathological conditions and neurodegenerative diseases (Kimelberg and Nedergaard, 2010). Neurons have a lower antioxidant capacity than astrocytes and, therefore, are more susceptible to damage, requiring stronger metabolic coupling to antagonism oxidative stress both under normal conditions and in cases of brain damage (Hamby and Sofroniew, 2010). Antioxidative protection of neurons, NTFs, and substrates required for neuronal metabolism, and reuptake of glutamate can be supported by astrocytes (Greve and Zink, 2009; Barreto et al., 2011b). When astrocytes are severely damaged, neurons die; postmortems of the brains of patients with PD have demonstrated increased astrocyte reactivity, interferon-gamma and neurotrophic factor release, glutathione peroxidase (GPx) levels, and the endocytosis of α-synuclein by glial cells (Chung et al., 2010).

Astrocytes respond to brain injury, including oxidative stress in neurodegeneration through the process of “reactive astrogliosis” (Barreto et al., 2011a,c, 2012). This involves changes at the molecular level, including the increased expression of glial fibrillary acid protein (GFAP), vimentin, nestin, and the Ras homologous protein (RhoA). Oxidative stress is prevented by increasing glutamate uptake to produce glutathione, releasing adenosine to protect neurons, degrading β-amyloid peptides, regulating the BBB, and forming glial inclusions. In addition, reactive astrocytes can release inflammatory cytokines (including TNFα) and ROS (Sugaya et al., 1998; Fitch and Silver, 2008; Duffy et al., 2009; Hamby and Sofroniew, 2010; Kang and Hebert, 2011). Astrocyte proliferation may play conflicting roles during PD episodes. For example, it has been reported that an increase in the number of reactive astrocytes plays a role in dopaminergic neuron repair (Chen et al., 2005; Francesca et al., 2013), yet reactive astrocyte content is reduced in the pathological tissue of patients with PD after death. This implies that the excessive accumulation of α-synuclein can inhibit astrogliosis and exert neuroprotective effects (Song et al., 2009; Tong et al., 2015). Irreversible changes in the cytoskeleton of astrocytes can be caused by the inactivation of RhoA by the botulinum C3 toxin, resulting in astrocytic morphological stellation associated with actin and intermediate filament disassembly (Ramakers and Moolenaar, 1998). It has also been reported that increases in peroxide and NO levels induce the activation of Rho/Rho-associated protein kinase (ROCK) based on a vascular model (Noma et al., 2007; Kagiyama et al., 2010). The activation of RhoA in endothelial cells during angiogenesis could be caused by PDGF-BB (Nobes et al., 1995; Amerongen et al., 2003). In this regard, Fujimura and Usuki (2012) confirmed that exposure to 100-nM rotenone or inorganic mercury suppressed the expression of cell division control protein 42 (CDC42) and Rac1 without affecting the expression of RhoA, and this process was accompanied by axonal degeneration and cortical brain cell death (Masatake and Fusako, 2012). Similarly, the pharmacological inhibition of ROCK reduces ERK1/2 phosphorylation even after stimulation of glioblastoma cells with PDGF-BB (Zohrabian et al., 2009). A seminal study by Richardson et al. (1988) demonstrated that PDGFs stimulated growth of cultured OPCs from rat optic nerves (Richardson et al., 1988). Subsequent studies using *in vivo* experiments in mice confirmed that OPCs can express PDGFR-α, and PDGF-AA was found to promote OPC proliferation (Fruttiger et al., 1996, 1999; Calver et al., 1998). *In vivo* astrocytes and neurons are able to stimulate OPCs through the paracrine release of PDGF (Fruttiger et al., 2000), with PDGF-AA appearing to be a determinant of OPC proliferation rates (Woodruff et al., 2004). Additionally, Huang et al. (2014) found that matrix-derived PDGF-C is a key factor in the recruitment and activation of oligodendrocyte progenitors (Huang et al., 2014).

As the primary immune cells of the CNS, microglia are important in host defense against invading microorganisms and tumor cells. Microglia may also play a dual role in immune responses, protecting the CNS by amplifying inflammatory responses and mediating cellular degeneration (Gonzalez-Scarano and Baltuch, 1999). Importantly, the pathogenesis of neuroinflammation and neurodegenerative diseases including PD may be related to the activation of microglia. It was also found that microglial cells highly express PDGFRβ, the receptor of αVβ3 integrin molecules, the binding of which contributes to tissue regeneration, angiogenesis, and tumor metastasis (Schneller et al., 1997). This was confirmed in a subsequent study in mouse microglial cells, where inflammation was suppressed by the interaction between the microglial PDGFRβ receptor and the αVβ3 integrin complex of the MBP-primed Th2 cells (Roy and Pahan, 2013).

#### Platelet-Derived Growth Factors Regulate Neuroglobin Expression

Ngb has been shown to perform neuroprotective functions by targeting neurons and astrocytes during pathological processes, such as focal ischemia, AD, strokes, and traumatic brain injury (Emara et al., 2009; Chen et al., 2015; Avila-Rodriguez et al., 2016; Xie and Yang, 2016). Furthermore, studies have shown that PDGF-BB can upregulate cytoglobin expression in hepatic stellate cells as well as the expression of GPx1 and Ngb in an astrocyte model during rotenone injury. This indicates that PDGF-BB may play a neuroprotective role in PD by regulating Ngb expression (Cabezas et al., 2018).

#### Platelet-Derived Growth Factors Regulate Pericyte Abundance

Pericytes are important for regulating blood pressure and the structural integrity of the blood vessel wall. Pericyte dysfunction triggers the breakdown of the BBB, leading to the accumulation of toxic proteins in the brain and a reduction in cerebral blood flow, which, in turn, reduces the delivery of nutrients and oxygen to the brain, leading to secondary neurodegeneration (Sagare et al., 2013).

Matrix metalloproteinases (MMPs) are zinc-dependent endopeptidases involved in the breakdown of the extracellular matrix in normal physiological processes and disease processes, especially in PD (Yong, 2005; Cauwe and Opdenakker, 2010). As an important member of MMPs, MMP-9 is upregulated in PD (He et al., 2013). Among the BBB-constituting cells, brain pericytes were found to be the most MMP-9-releasing cells in response to thrombin stimulation (Machida et al., 2015). Furthermore, the overexpression of α-synuclein stimulates MMP-9 activity (Lee et al., 2010), while neuronal cell death caused by the dopaminergic neurotoxins 6-OHDA (6-hydroxydopamine) and MPP (+) can be ameliorated by the administration of MMP-9 inhibitors (Joo et al., 2010). Machida et al. (2017) found that thrombin activates two independent signaling pathways by acting on PAR-1 expressed by brain pericytes (the PKCθ-Akt and PKCδ-ERK1/2 pathways) and causes brain pericytes to release MMP-9, which leads to BBB dysfunction (Machida et al., 2017). Dohgu et al. (2019) confirmed that pericytes are also capable of releasing various inflammatory cytokines/chemokines in response to monomeric α-synuclein to induce BBB dysfunction, thus contributing to the progression of PD (Dohgu et al., 2019). In pericytes, tunneling nanotubes (TNTs) function as F-actin-based membranous channels, which connect cells and contribute to cell-to-cell transmission of α-synuclein (Dieriks et al., 2017).

PDGF-B plays a crucial role in the recruitment of pericytes to various vascular beds, including the brain, kidneys, heart, lungs, and adipose tissue (Brownlee, 2001), and recruited pericytes are reduced in number in the absence of the PDGF-B allele (Benjamin et al., 1998). Tallquist et al. (2003) found that the number of pericytes in heterozygous PDGFR-β mice was reduced, which also suggests that the number of pericytes may be related to PDGFR-β expression (Tallquist et al., 2003). PDGFR-β has also been found to act as a non-specific diagnostic marker for pericytes (Armulik et al., 2011). Gene ablation of PDGF-B or PDGFR-β in mice was also found to result in almost identical phenotypes, namely, perinatal death following extensive microvascular leakage and hemorrhage (Leveen et al., 1994). Severe pericyte deficiency also causes microvascular dysfunction in these mice, and while pericytes appear to be induced in the absence of PDGF-B or PDGFR-β, subsequent selection (expansion and spread) of pericyte populations fails due to reduced pericyte proliferation. The migration of pericytes along new vessels may also be impaired when PDGF-B/PDGFR-β signaling is disrupted. In angiogenesis, PDGF-B is expressed by sprouting endothelial cells, whereas PDGFR-β is expressed by pericytes/VSMC precursor cells (Lindahl et al., 1997; Hellström et al., 1999). This suggests that the interaction between these two cell types is a paracrine stimulation. In chimeras consisting of PDGFR-β-positive and PDGFR-β-negative cells, only PDGFR-β-positive cells were found to aggregate between VSMCs/pericytes, suggesting that the development of these cells is directly dependent upon on PDGFR-β. In addition, Enge et al. (2003) found that the knockout of the endothelial-specific PDGF-B gene results in VSMC/pericytic defects (Enge et al., 2003). Gene ablation of PDGF-B in hematopoietic cells or neurons (the other two major sources of PDGF-B) has no apparent effect on the vasculature, and the available evidence confirms that endothelial PDGF-B signaling controls pericyte recruitment during angiogenesis. The number of pericytes (or progenitors) that can be recruited may depend on the amount of PDGF-B available (van Heyningen et al., 2001). Notably, the effect of PDGF-B on mature vasculature is dose dependent (Ejaz et al., 2008). Padel et al. (2016) found that a 2-week treatment with PDGF-BB promoted the recovery of behavioral function and partially restored the nigrostriatal pathway. At the same time, pericytes in the striatum of PD model mice were activated, and this change could be reversed by PDGF-BB treatment. This demonstrates that brain pericytes may play a role in the pathogenesis of PD and may be a target for PDGF-BB treatment of PD neural recovery mechanisms (Padel et al., 2016). Subsequently, using an *in vitro* model of dopaminergic injury, Gaceb et al. (2020) demonstrated that PDGF-BB/PDGFRβ-mediated brain pericyte secretion affects the expression of dopamine markers, which may shed light on the mechanism by which PDGF-BB promotes neural recovery in PD (Gaceb et al., 2020). These studies provide inspiration for future related research in this field.

## Platelet-Derived Growth Factors for the Treatment of Parkinson’s Disease

The treatment of PD is currently mainly limited to symptomatic treatments, such as the use of drugs levodopa, carbidopa, and dopamine receptor agonists, as well as catechol-O-methyltransferase (COMT), monoamine oxidase (MAO-B) inhibitors, and deep brain stimulation. The aim of treatment is primarily to maintain or prolong the patient’s daily activities without slowing or reversing the progression of PD. However, these treatments tend to lose their efficacy after 2–5 years (Hauser et al., 2006; Bronstein et al., 2011; Post et al., 2011; Chmielarz and Saarma, 2020). Therefore, there is an urgent need to identify effective and long-lasting neuroprotective agents to avoid further degeneration of nigrostriatal neurons and axons, thereby slowing the development of disease. Decreased levels of NTFs and knockdowns of their receptors have been reported to trigger neuronal loss and other outcomes related to disease progression (Rinne et al., 1989; Lorigados Pedre et al., 2002). As an endogenous growth factor that boosts neuronal survival and differentiation, the therapeutic potential of PDGF has been validated in various neurodegenerative diseases, owing to its neuroprotective and neurorestorative properties. Upregulated expression of PDGF-A and PDGF-B mRNA, and PDGF-AA, PDGF-BB, and PDGF-AB proteins, as well as PDGFR-α and PDGFR-β receptors, has been observed in neuronal samples from patients with ischemia. This suggests that these ligands and their receptors may be important for neuronal survival in damaged brain regions. Indeed, the neuroprotective effects of exogenous PDGF-BB during focal ischemia have been confirmed in multiple studies (Krupinski et al., 1997; Sakata et al., 1998), and glutamate- and NMDA-induced neuronal death in the hippocampus may also be blocked. Lesions caused by NMDA hyperstimulation in neonatal rats can be decreased by intracerebral administration of PDGF-BB (Egawa-Tsuzuki et al., 2004; Tseng and Dichter, 2005), and pretreatment with PDGF-BB at doses of 120–240 ng/ml has been demonstrated to reduce pyramidal neuron death during ischemic injury in rats (Iihara et al., 1997). Damage to the striatal dopaminergic system *in vivo* also provides evidence of elevated PDGF-B levels, which may reflect the endogenous neuroprotective effects of PDGF-B (Funa et al., 1996). Based on a rodent model of PD, Zachrisson et al. (2011) demonstrated that intracerebroventricular administration of PDGF-BB provides an alternative strategy for restoring function in PD. According to their animal model of nigrostriatal injury, the administration of PDGF-BB treatment for 2 weeks resulted in the increased expression of striatal dopamine transporter binding sites and SN TH, normalizing amphetamine-derived rotational behavior in 6-OHDA-injured rats. The same authors showed that PDGF-BB can also promote the proliferation of NPCs in the subventricular zone, and, when co-infused with a proliferation inhibitor, can block the recovery of dopaminergic neuron function. This work suggests that PDGF-BB has a restorative effect on 6-OHDA- and MPTP (1-methyl-4-phenyl-1,2,3,6-tetrahydropyridine)-injured rat dopaminergic neurons, and, further, application in human clinical trials did not produce adverse effects (Zachrisson et al., 2011).

The effects of PDGF-BB on dopaminergic neurons are the result of persistence rather than direct pharmacological effects. In a PD mouse model with partial 6-OHDA medial forebrain tract lesions, along with the restoration of the nigrostriatal pathway and the inhibition of pericyte activation, PDGF-BB was found to guard against behavioral disorders (Padel et al., 2016). Chao et al. (2014) also confirmed that PDGF-BB ameliorates human immunodeficiency virus-1 (HIV-1) transcriptional transactivator (Tat) levels by activating p38 and N-terminal kinase/mitogen-activated protein kinase (JNK/MAPK) pathways, thereby impairing the proliferation of NPCs, which are specialized cells with the potential to develop into neurons during neurogenesis. The researchers also reported that the novel GSK-3β/β-catenin pathway is involved in neurogenesis mediated by PDGF-BB; as the main substrate of GSK-3β, the level of nuclear β-catenin increased in the presence of PDGF-BB, indicating its potential stimulating effect on NPC proliferation (Chao et al., 2014). In rat hippocampal neurons, PDGF-BB regulates Arc/Arg3.1 gene expression by activating the MAPK/ERK pathway and, therefore, affects synaptic plasticity and long-term potentiation (Peng et al., 2008). Recently, it has been reported that neuroprotection in PD animal models can be induced by PDGF-CC levels and their signaling (Tang et al., 2010). Oxidative stress, neurotoxin production, and apoptosis can be inhibited, modulating GSK-3β activity *in vivo* and *in vitro via* PDGF-C, thus acting on various neuronal cell types. In contrast, mice lacking PDGF-CC expression show an increased rate of neuronal death (Tang et al., 2010). Peng et al. (2012) demonstrated that PDGF-CC can protect mouse neuronal cells from apoptosis caused by different types of toxins containing 6-OHDA and HIV TaT, and can also activate TRPC Channel 1 to prevent the production of HIV TaT toxin, which regulates downstream protein pathways, such as the GSK3β pathway in the SH-SY5Y neuroblastoma cell line (Peng et al., 2012). Additionally, serotonin receptor agonists have also been shown to block NMDA-induced cell death by increasing PDGFR-β expression in primary hippocampal neurons (Vasefi et al., 2012, 2013). Studies have shown that Notch3-/- mice have reduced PDGFR-β levels, which indicates that the PDGFR-β signaling pathway may interact with Notch signaling, thereby jointly affecting neurodegenerative diseases (Jin et al., 2008; Nadeem et al., 2020).

The safety and the tolerability of intraventricular recombinant human PDGF-BB (rhPDGF-BB) administration in patients with PD were evaluated in a double-blind, randomized controlled trial by Paul et al. (2015). The results demonstrated that all doses of rhPDGF-BB were well-tolerated by the human body; RhPDGF-BB had no unresolved adverse effects on the patients with PD and a positive effect on the binding of dopamine transporters in the right putamen (Paul et al., 2015). At present, the therapeutic effect of PDGFs on PD has been verified in cell and animal experiments, but relevant clinical trials are still lacking, and the application of PDGFs in PD still faces ethical and immuneresponse-related questions. Table 1 summarizes existing trials on the safety and efficacy of PDGFs for the treatment of certain diseases, including PD. Nevertheless, their application in clinic settings still faces many problems. First, in terms of performance, the clinical benefits of PDGFs are presently still low. Second, the dose of PDGFs delivered to the brain is uncertain (Bartus and Johnson, 2017); PDGFs also have a short half-life *in vivo* and poor pharmacokinetic properties, and the permeability of PDGFs through the BBB is very low. It is, therefore, necessary to identify intracranial delivery routes.

**TABLE 1:** Clinical trials of platelet-derived growth factors (PDGFs) for disease treatment.

PDGF	Clinical trials	References
PDGF	It was confirmed that the PDGF purified from human placenta (EAP) can induce tritiated thymidine incorporation in Chinese hamster lung fibroblasts (CCL39).	(Marez et al., 1987)
PDGF	Autologous adipose tissue grafts for human immunodeficiency virus facial lipoatrophy achieved better results without the addition of PDGFs.	(Fontdevila et al., 2014)
PDGF	This clinical experiment confirmed that PDGF/IGF-1 can promote periodontal regeneration.	(Giannobile et al., 1994)
PDGF	This clinical trial demonstrated that PDGF can promote periodontal regeneration in localized bone defects.	(Nevins et al., 2013)
PDGF	PDGF can be used as adjunctive treatment for pressure ulcers; preoperative treatment with rhPDGF-BB showed a greater ability to heal wounds than surgery alone.	(Kallianinen et al., 2000)
rhPDGF	This clinical trial evaluated the effect of calcium hydroxide as a matrix carrier for recombinant human PDGF on pulp tissue healing after pulp capping.	(Al-Hezaimi et al., 2020)
PDGF inhibitor	The combined intravitreal injection of ranibizumab (a vascular endothelial growth factor inhibitor) and E10030 (a PDGF inhibitor) was preliminarily shown to be safe, but its therapeutic efficacy remains limited.	(Hwang et al., 2021)
PDGF inhibitor	In this phase IIb clinical trial, PDGF antagonist E10030 was administered in combination with the anti-vascular endothelial growth factor drug ranibizumab (Lucentis) in the treatment of neovascular age-related macular degeneration, showing a favorable safety and efficacy profile.	(Jaffe et al., 2017)
PDGF-B	In this phase I trial, the safety of H5.020cmv.pdgf-B was evaluated for the treatment of diabetic insensitive foot ulcers.	(Margolis et al., 2000)
PDGF-BB	Intra-arterial injection of bone marrow mononuclear cell in patients with subacute stroke induced changes in serum levels of PDGF-BB, which may be related to prognosis.	(Moniche et al., 2014)
PDGF-BB	This clinical trial demonstrated that PM coverage of periodontal defects was associated with the upregulation of initial gingival crevicular fluid growth factors, which could improve surgical outcomes.	(Gamal et al., 2016)
PDGF-BB	This clinical trial demonstrated that purified recombinant human platelet-derived growth factor-BB/beta-tricalcium phosphatecan be used as an effective autograft substitute.	(DiGiovanni et al., 2013)
PDGF-BB	rhPDGF-BB + β-TCP is safe and effective in the treatment of periodontal defects, increasing bone formation and soft tissue healing.	(Jayakumar et al., 2011)
PDGF-BB	This clinical trial validated the safety and efficacy of rhPDGF-BB for the treatment of periodontal bone defects.	(Nevins et al., 2005)
PDGF-BB	Topical application of rhPDGF-BB and (rh)insulin-like growth factor-I to periodontal lesions was found to be safe and promote bone regeneration.	(Howell et al., 1997)
PDGFR-α	This clinical trial demonstrated that patients with high pretreatment anti-PDGFRA antibody levels raise the risk-to-benefit ratio of nilotinib.	(Chen et al., 2018a)

The degeneration of nigrostriatal dopaminergic neurons is the main pathogenic characteristic of PD. A feasible treatment may be the local delivery of therapeutic proteins with neuroprotective and restorative properties into the neuronal pathways projecting to the dorsal striatum *via* axonal transport through dopaminergic neuron cell bodies in the SNpc. Additionally, the effects of PDGFs on neuronal therapeutic targets may be limited by other factors, such as the degradation of the PDGF protein itself, the role of clearance mechanisms *in vivo* (such as liver and kidney metabolism), and the possible combination with various components of peripheral tissues (Thorne and Frey, 2001). Invasive treatments are considered unethical for patients in the early stages of PD, and PDGFs can only be delivered directly to the patient’s brain through intracranial surgery. For this reason, it is particularly important to develop an efficient peripheral PDGF delivery system for alternative therapies (Carl-Henrik and Bengt, 1999).

### Treatment of Parkinson’s Disease Based on the Interaction of Stem Cells and Platelet-Derived Growth Factors

In recent years, research on stem cells in the treatment of neurodegenerative diseases, including PD, has made great progress. Preclinical studies have shown the potential of mesenchymal stem cells (MSCs) for neural transplantation, as this subtype of stem cells is able to migrate to sites of damaged neural tissue, following bone marrow-derived MSCs or amniotic fluid-derived stem cells by intravenous and intracranial transplantation (Li et al., 2001; Chopp and Li, 2002; Hellmann et al., 2006; Cipriani et al., 2007). As a type of MSCs, human endometrial-derived stem-like cells (HEDSCs) have been characterized, which can transdifferentiate into cartilage, bone, fat, and muscle *in vitro* (Schwab and Gargett, 2007; Gargett et al., 2009). It has also been confirmed that HEDSCs are able to differentiate into dopamine-producing neurons and have the ability to migrate. *In vivo*, HEDSCs can be transplanted, they migrate to the diseased site, and differentiate spontaneously. The therapeutic benefits of increasing dopamine concentrations in a mouse model of immunocompetent PD have been also demonstrated based on flow cytometry and HEDSC characterization, which were strongly positive for both PDGF-Rβ and CD146 (Wolff et al., 2011).

Ebrahimi-Barough et al. (2013) exposed endometrial stromal cells to growth factors and mitogen PDGF-AA and obtained oligodendrocyte progenitor cells. Their RT-qPCR results verified that these cells expressed OPC markers, including PDGFRα. Furthermore, mir-338 was successfully used to promote oligodendrocyte differentiation of human endometrial-derived stromal cell (hEnSC)-derived OPC, and the data suggest that these cells can be differentiated into the pre-oligodendrocyte phenotype *in vitro* (Ebrahimi-Barough et al., 2013).

Pelegri et al. (2019) found that 100-ng/ml VEGF/PDGF had the greatest effect on the proliferation of hippocampal neural stem cells (HNSC), and the differentiation pathway induction in this treatment group showed the most significant oligodendrocyte and neuronal markers and morphological characteristics (Pelegri et al., 2019). These promising findings should be verified in further studies on brain and spinal cord injuries, including PD. Additional studies showing that PDGF interacts with stem cells to affect cell function are summarized in Table 2.

**TABLE 2:** A summary of the interaction between platelet-derived growth factors (PDGFs) and stem cells.

PDGF	Effect on stem/Progenitor cells	References
PDGF	Stem cells (human adipose-derived stem cells) incorporating PDGF and organisms (biological mineral coated fibers) can be used to successfully regenerate vascularized bones.	(Lee et al., 2020)
PDGF	PDGF can regulate extracellular vesicles of adipose-derived mesenchymal stem cellsto regulate protein expression and their functions.	(Lopatina et al., 2018)
PDGF	Hematopoietic stem cells overexpressing PDGF for regenerative therapy are beneficial for the improvement of myocardial function in rats, while the level of tissue connexin 43 and proangiogenic molecules increased after infarction.	(Das et al., 2009)
PDGF	The differential activation of phospholipases probably is significant for neurotrophic PDGF in HiB5 neuronal hippocampal stem cells. Neuronal differentiation by neurogenic PDGF in the HiB5 cells may be regulated by the activation of phospholipase C and D.	(Sung et al., 2001)
PDGFs	Platelet-rich plasma (PRP) immobilized on gelatin microspheres (GMs) by a mussel-inspired polydopamine (GM-pDA-PRP) was used for creating a microenvironment for the proliferation of adipose-derived stem cells. PDGF prolonged and localized production was induced by enhanced PRP adhesion.	(Zhou et al., 2017)
PDGF-AA	PDGF-AA and expression of exosome CD81 and CD9 can be secreted by cell-free stem cell-derived extract (CCM) formulated from human progenitor endothelial stem cells (hPESCs). CCM promoted cell proliferation and induced stem cell migration.	(Gupta et al., 2020)
PDGF-AA	Tppp3 + PDGFRA + cells are equivalent to tendon stem cells. Tppp3-PDGFRA + fibro-adipogenic progenitors coexist in the tendon stem cell niche and promote the production of fibrotic cells.	(Harvey et al., 2019)
PDGF-AA/PDGFRα	In mesenchymal stem cells (MSCs), PDGF-AA was found to activate the BMP-Smad1/5/8 pathway, which requires BMPRIA and PDGFRα together to promote MSC osteogenic differentiation and MSC migration.	(Li et al., 2014)
PDGFR-α	In zebrafish, trunk neural crest migration to the dorsal aorta is required for hematopoietic stem cell specification, which is regulated by PDGF signaling.	(Damm and Clements, 2017)
PDGF-AB PDGF-BB	The effectiveness of human serum on human adipose-derived stem cellproliferation depends on the concentrations of endogenous PDGFs.	(Damm and Clements, 2017)
PDGF-BB	Co-overexpression of PDGF-BB and IL-4 was found in co-infected MSCs, which promote cell proliferation and viability, as well as osteogenesis.	(Zhang et al., 2021)
PDGF-B	Gene embedded (pDNA-platelet-derived growth factor, PDGF-B) porcine acellular urinary bladder matrix with transfected mesenchymal stem cells can release PDGF-B, which promotes neovascularization and new tissue formation. The secretion of other growth factors was promoted by the expression of PDGF, leading to PDGF-mediatedregenerative activity.	(Paramasivam et al., 2021)
PDGF-BB	PDGF-BB-treated cells were associated with the endothelial network and expressed markers of perivascular cells while also promoting satellite cell self-renewal. The treated cells obtained the ability to migrate across the endothelium.	(Gerli et al., 2019)
PDGF-BB	The proliferation of mesenchymal stem cells in human periodontal ligament was promoted by PDGF-BB.	(Mihaylova et al., 2018)
PDGF-BB	PDGF-BB promoted fibroblast growth in factor 2 mouse embryonic stem cell conditioned medium (mESC-CM), which is important for the antisenescence effect of mESC-CM.	(Bae et al., 2016)
PDGF-BB	PDGF-BB promoted 3D-encapsulated mesenchymal stem cellsdose-dependent proliferation, spreading, and migration.	(Lienemann et al., 2015)
PDGF-BB	Treatment with PDGF-BB activated Akt phosphorylation, decreased p53 expression, and reduced radiation-induced apoptosis in mouse intestinal progenitor/stem cell.	(Liu et al., 2014)
PDGF-BB	PDGF-BB promoted the proliferation of human mesenchymal stem cells.	(Dong et al., 2015)
PDGF-BB	Membrane sections with higher PDGF-BB concentrations created a better environment for human adipose-derived stem celltenogenesis.	(Min et al., 2014)
PDGFR-β	The activation of PDGFR-β contributed to vascular smooth muscle cell differentiation.	(Shimizu et al., 2008)
PDGFR-β	PDGFR-β signaling pathways are involved in the differentiation of embryonic stem cells into smooth muscle cells.	(Xiao et al., 2007)

The application of embryonic stem cells (ESCs) for PD therapy has been validated by multiple studies, yet the use of ESCs faces difficult ethical issues due to access and application methods. The discovery of the induced pluripotent stem cell (iPSC) technology has greatly helped address these problems. Specifically, numerous studies have demonstrated that iPSCs and ESCs are molecularly and functionally equivalent, avoiding the ethical issues and the constraints of traditional methods. This provides a meaningful solution for a sustainable source of pluripotent stem cells without the need for immunosuppressive agents to combat immune rejection after implantation therapy (Yefroyev and Jin, 2022). Similar to ESCs, iPSCs show self-renewal ability and multi-directional differentiation potential. In recent years, iPSCs and various neurons derived from them have been used in the treatment of neurodegenerative diseases, including PD. Rhee et al. (2011) demonstrated that the behavioral deficits in a rodent model of PD were significantly ameliorated by dopamine neurons derived from protein-based hiPSCs (human-induced pluripotent stem cells) (Rhee et al., 2011). Hartfield et al. (2014) also confirmed that hiPSC-induced dopaminergic neurons can synthesize, secrete, and reabsorb dopamine (Hartfield et al., 2014). Song et al. (2020) have recently developed a more efficient method for generating clinical-grade iPSCs by combining metabolic-regulating microRNAs with reprograming factors. The induced cells exhibit the electrophysiological characteristics of dopamine neurons, and these researchers reported that their transplantation into a PD rodent model potently restores motor function and reactivates the host brain, helping progress toward human personalized autologous cell therapy for PD (Song et al., 2020). The study by Kikuchi et al. (2017) demonstrated that human iPSC-derived dopaminergic progenitor cells survive and function as midbrain dopaminergic neurons in a PD primate model (a cynomolgus monkey) treated with the neurotoxin MPTP (Kikuchi et al., 2017). In addition, secondary changes in the GABAergic nervous system can also directly or indirectly affect the pathogenesis of PD (Mochizuki et al., 2008). Studies have also demonstrated that human-derived iPSCs can be directly induced into GABAergic neurons, thus providing candidate cells for PD therapy (Liu et al., 2013). However, while iPSCs can replace patient-specific diseased cells, this approach is not without limitations. For example, genomic instability and epigenetic aberrations must be considered, which may be caused by reprograming (Weissbein et al., 2014). Furthermore, due to the low degree of differentiation of iPSCs, once out of control, they may form difficult-to-treat tumors. At present, there is a lack of clinical research on the relationship between iPSCs and PDGFs, and their possible combined role in PD generation is unknown.

### Parkinson’s Disease Treatment Based on the Interaction of Genes and Platelet-Derived Growth Factors

Several studies have shown that PDGFs can be combined with other cells, carriers, cytokines, and biological materials to deliver effects to the injury site (Johnson et al., 2010). In terms of genes, Liu et al. (2011) constructed the hS100B transgenic vector by inserting the human *S100B* gene downstream to the PDGF promoter, which was then microinjected to generate transgenic mice. They confirmed that S100B overexpression in the brain leads to a motor coordination disorder, which may be related to the downregulation of D2DR and GRK2 expression, increased dopamine anabolism, and reduced 5-HT levels (Liu et al., 2011).

As a rapidly developing technology, the efficient delivery of genetic material in neurons using viral vectors is a key approach of gene therapy, thus modulating the expression of one or more specific genes. Gene therapy for PD has undergone three significant changes in the past two decades (Bjorklund and Davidsson, 2021), from *ex vivo* gene transfer using retroviral vectors to *in vivo* gene expression using adenovirus, herpes simplex virus (HSV), or lentiviral vectors. Recently, a major breakthrough has been made in the application of adeno-associated virus (AAV) vectors in CNS gene therapy, involving the repair of dopamine synthesis, strengthening of trophic factor production and lysosomal function, or alteration of the interactions between different functional nodes of the basal ganglia, including clinical trials for PD (Merola et al., 2020).

In the case of PD gene therapy, AAV capsid engineering shows some promise. For example, an AAV-2 vector containing a hybrid CMV E/PDGF promoter was developed and found to be superior in driving gene expression in SN dopaminergic neurons (Wang et al., 2005). However, not every cell surface can be targeted for AAV-engineered therapy, making precise and specific regulation of each target difficult. Although gene therapy is generally irreversible, in some cases, it can be modulated by modulating the inflammatory state and the cascade of apoptosis. Earlier approaches to inserting cell-type-specific promoters into the AAV genome to drive its transgene expression led to disappointing results with unsatisfactory specificity (Mudannayake et al., 2016). Current sequencing technologies allow the addition of short enhancer sequences upstream of minimal promoters, which helps accurately identify short enhancer elements with specificity. This approach was successfully used to identify enhancer elements associated with PD (Graybuck et al., 2021). Relevant dopamine neuron subtypes and disease states can be identified by the AAV vector, enabling precise-targeted expression of therapeutic transgenes, avoiding immune responses, and reducing the dose required. Engineered AAV vectors can be combined with cell therapy from exogenous cell sources, such as ESC or IPS cells, to facilitate dopamine engraftment for enhanced therapeutic efficacy. Therefore, AAV engineering has the potential to radically improve target specificity (Xiong et al., 2021).

In a future where autologous cell transplantation (including reprograming) is commonplace, any disease-causing genetic changes in host cells may be treated by viral vector engineering (e.g., alpha-synuclein overexpression) (Stoddard-Bennett and Reijo Pera, 2019). Such therapies can target macroautophagy, slow neuronal aging, and rejuvenate dopamine neurons, even in presymptomatic PD or in people at high risk of developing PD (Benito-Cuesta et al., 2017; Lu et al., 2020; Minhas et al., 2021). Therefore, genetically targeted therapy could be used for neuroprotection and the prevention of neurodegenerative diseases in the wider population. In addition, delivery strategies based on nanoparticles (NPs) can improve the stability of NTFs and, thus, have potential for clinical applications. NPs have been confirmed to function as non-viral vehicles to deliver drugs to different cells and organs (Oberdörster et al., 2007). The advantage of this approach is that they can passively accumulate in specific cells by improving the stability and physicochemical properties of active drugs. However, most of the current approved nanomedicines are cancer drugs; the application of NPs in PDGF-replacement support therapy for PD may become an important clinical strategy to slow down or even reverse disease progression in the future (Bondarenko and Saarma, 2021).

### Parkinson’s Disease Treatment Based on the Interaction of Epigenetic Modifications and Platelet-Derived Growth Factors

Epigenetics refers to changes in gene expression or function that occur without changes to the DNA sequence. This plays an important role in regulating neural development, neural stem cells, and the physiological functioning of the nervous system. In recent years, epigenetic modification, as the link between heredity and the environment, has gradually attracted attention for its relationship with the pathogenesis of PD (Jasiulionis, 2018). Epigenetic modifications include DNA methylation, DNA hydroxymethylation, histone modifications, and non-coding RNA (ncRNA)-mediated changes in gene expression.

It has been confirmed that DNA methylation can regulate the PD pathogenic gene *SNCA* (Matsumoto et al., 2010). Based on Clustered Regularly Interspaced Short Palindromic Repeats (CRISPR)-deactivated Cas9 (dCas9) fused with the catalytic domain of DNA-methyltransferase 3A (DNMT3A), Kantor et al. (2018) targeted methylation of *SNCA* intron 1 using a lentiviral vector, which resulted in the downregulation of SNCA mRNA and its protein in PD patient-derived neurons, thereby alleviating associated cellular phenotypic features, including mitochondrial ROS production and cellular activity (Kantor et al., 2018). In addition, the *MAPT* gene is associated with the pathogenesis of PD because it encodes the microtubule-associated protein tau. Different *MAPT* methylation levels have been observed in frontal cortex and peripheral blood leukocytes between patients with PD and the controls (Masliah et al., 2013). Marshall et al. (2020) found that more than 70% of 1,799 differentially methylated sites of enhancers in neurons of patients with PD had elevated methylation levels. Among the 2,885 genes involved, these researchers identified many risk genes related to the pathogenesis of PD, including *DCTN1*, *PRKN*, and *DJ-1*, and showed that the inactivation of the *TET2* gene can have a therapeutic effect on PD by reducing neuroinflammation (Marshall et al., 2020). Han et al. (2014) demonstrated that VSMC proliferation can be caused by the epigenetic regulation of PDGFs; they found that homocysteine induces the hypomethylation of the PDGF gene promoter region and upregulates its mRNA and protein expression (Han et al., 2014). However, the specific roles of PDGF methylation in PD need to be explored in the future.

In recent years, the DNA hydroxymethylation epigenetic modification has gradually attracted attention, as its correlation with neurodegenerative diseases has been verified (Sherwani and Khan, 2015). Studies have shown that, in patients with PD, the median level of the hydroxylated form of 5-methylcytosine, 5-hydromethylcytosine (5hmC), almost doubles that in controls (Stöger et al., 2017). This suggests that DNA hydroxymethylation is associated with the pathogenesis of PD, although there is still a lack of research in this area. Thus far, no research has focused on the DNA hydroxymethylation of PDGF genes.

Histones are octamers composed of H2A, H2B, H3, and H4, with tail residues presenting various types of post-transcriptional modifications, including methylation, acetylation, phosphorylation, ubiquitination, ADP ribosylation, deamination, proline isomerization, and lysine threoninylation. Studies have shown that histone acetylation levels are significantly elevated in midbrain dopaminergic neurons of patients with PD (Park et al., 2016). Curcumin, a histone deacetylase (HDAC) inhibitor, was found to reduce apoptosis and improve motor deficits in a DJ-1 knockout rat model of PD (Chiu et al., 2013). HDAC inhibitors are also able to reduce neuroinflammation by reducing microglial activation, thereby protecting dopaminergic neurons. On the other hand, as the main cause of autosomal dominant familial PD, the pathogenic effects of *LRRK2* mutations are closely related to histone modification. *LRRK2* can bind to the ser424 site of HDAC3 and directly phosphorylate HDAC, thereby stimulating its activity and promoting histone H4 deacetylation, resulting in gene transcriptional repression. In addition, increased nuclear DNA damage and abnormal histone methylation were observed in striatal neurons of *Lrrk2*-/-aged mice (Chen et al., 2020). Histone modifications are also present in certain neural factors promoting dopaminergic neuron growth. These include modifications in the promoter regions of glial cell line-derived neurotrophic factor (GDNF) and brain-derived neurotrophic factor (BDNF). A variety of HDAC inhibitors can also cause upregulation of GDNF and BDNF gene expression (Yue et al., 2014). Owens (2007) showed that phenotypic switching of smooth muscle cells (SMCs) in response to PDGF-BB *in vitro* is ended, owing to the loss of a subset of activating histone modifications at gene loci encoding SMC marker genes (Owens, 2007). This may be enlightening for the research related to PDGF histone modifications.

m^6^A methylation is the most prevalent modification of eukaryotic mRNA. Foo et al. (2017) analyzed m^6^A-modified genes in 1,647 sporadic patients with PD and 1,372 controls. These authors found 214 rare mutations in all m^6^A-modified genes, and 16 common mutations were found in seven genes, implying that the pathogenesis of PD is related to m^6^A methylation (Foo et al., 2017). Studies have also shown that m^6^A reduction can induce the expression of NMDA receptor 1, increase oxidative stress and Ca^2+^ influx, and cause dopaminergic neuron apoptosis (Chen et al., 2019). At present, research on the role of m^6^A methylation in the pathogenesis of PD remains very limited. Zeng et al. (2021) showed that, compared with that in the controls, m^6^A methylation was increased in the lung tissues of a rat model with pulmonary arterial hypertension, and the up-methylated genes coding for the PDGF signaling pathway were primarily enriched (Zeng et al., 2021). Further research exploring the relationship between PDGFs and m^6^A methylation is needed.

**FIGURE 5:**
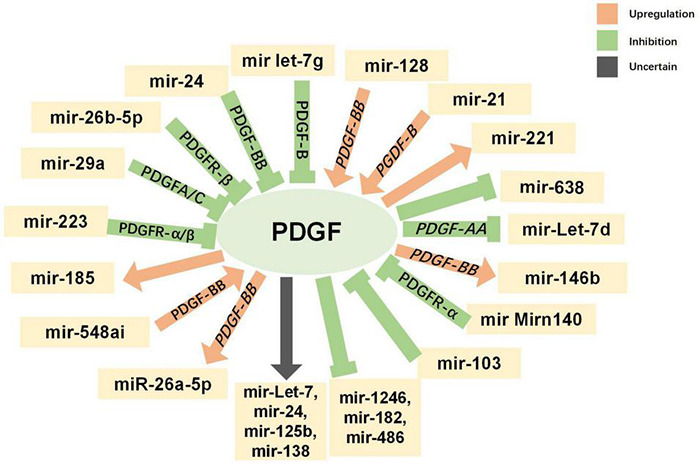
Interaction between platelet-derived growth factors (PDGFs) and microRNAs. Green indicates inhibitory regulation; orange indicates facilitative regulation; and gray indicates unclear regulation.

**FIGURE 6:**
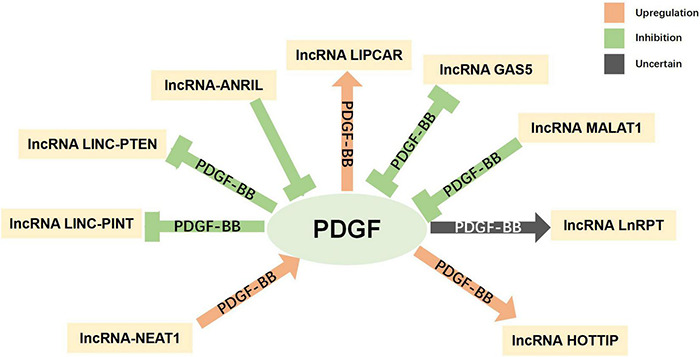
Interaction between platelet-derived growth factors (PDGFs) and long non-coding RNA. Green indicates inhibitory regulation; orange indicates facilitative regulation; and gray indicates unclear regulation.

Non-coding RNAs, including microRNAs (miRNAs) and long non-coding RNAs (lncRNAs), have been found to have varying expression levels in PD patients, and take part in regulating the pathogenetic mechanisms of PD (Roy et al., 2022). For example, miR-7 and miR-153 were proved to lower SNCA (synuclein α) expression levels (Junn et al., 2009; Doxakis, 2010), which is closely related to PD. Negatively controlled by LRRK2, miR-let-7 and miR-184 are involved in the reduction of dopamine levels in dopamine-producing cells (Gehrke et al., 2010). Both miRNAs and lncRNAs have been found to be diagnostic biomarkers for PD (Zhang et al., 2022). The interaction between PDGF and non-coding RNAs has been explored, especially in SMCs. MiR-24 can inhibit the proliferation and angiogenesis of VSMCs by suppressing the PDGF-BB signaling pathway (Yang et al., 2018). In PDGF-BB-stimulated airway smooth muscle cells (ASMCs), the expression of lncRNA PINT was reduced, while miR-26a-5p expression was increased (Gao et al., 2021). The current understanding of the interactions between non-coding RNAs (miRNA and lncRNA) and PDGF is summarized in Figures 5, 6 and more details are provided in Tables 3, 4. As the association between PDGFs and non-coding RNAs in PD treatment has not been explored, the information in these tables may help provide ideas for future research on PD-related treatments.

**TABLE 3:** Interaction between platelet-derived growth factors (PDGFs) and microRNA.

MicroRNA	Mutual effects of microRNAS and PDGF	References
miR-548ai	MiR-548ai inhibitor mitigates endothelial cell dysfunction induced by exosomes (PDGF-BB, TGFβ1, TNFα, and IL1β) derived from dysfunctional smooth muscle cells.	(Xie et al., 2021)
miR-185	The level of extracellular miR-185 increased in PDGF-stimulated vascular smooth muscle cells.	(Si et al., 2021)
miR-223	Overexpression of miR-223 alleviates GLI family zinc finger 2and platelet-derived growth factor receptor α/βexpression in hepatic stellate cells (HSCs), thus inhibiting the activation and proliferation of HSC.	(Wang et al., 2021)
miR-29a	At the transcriptional and translational levels miR-29a decrease the expressions of PDGFC and PDGFA.	(Yang et al., 2019b)
miR-26b-5p	MiR-26b-5p produces a negative adjustment of PDGFR-β and interacts with non-coding RNA maternally expressed gene (lncMEG3).	(Yang et al., 2019a)
miR-24	MiR-24 can reduce the expression of AP-1 by suppressing the PDGF-BB signaling pathway, thereby inhibiting the proliferation and angiogenesis of vascular smooth muscle cells.	(Yang et al., 2018)
miR let-7g	By targeting PDGF-B genes, miRlet-7g suppresses the phenotypic switching of vascular smooth muscle cells.	(Wang et al., 2017)
miR-21	Via miRNA-21-mediated PDCD4 downregulation, PDGF-BB stimulates cell proliferation and promotes the development of thyroid-associated ophthalmopathy.	(Lee et al., 2016a)
miR-221	By inducing miR-221, PDGFs affect both cell proliferation and the epithelial-mesenchymal transition phenotype, thus leading to the downregulation of p27Kip1 and TRPS1.	(Su et al., 2013)
miR-638	miR-638 expression was decreased in proliferative human VSMCs and its expression inhibited both SMC proliferation and migration in response to PDGF stimulation.	(Li et al., 2013)
miR-Let-7d, miR-146b, miR-638	In proliferative human VSMCs, miR-638 expression was decreased and both SMC proliferation and migration in response to PDGF stimulation were suppressed. PDGF-AA inhibited miRlet-7d, while PDGF-BB induced miR-146b in cancer cells. The induction of miR-146b by PDGF-BB is regulated via MAPK-dependent induction of c-fos.	(Shao et al., 2011)
miR-Let-7, miR-24, miR-125b, and miR-138	The basal characteristics of multipotent mesenchymal stromal cell differentiation into osteoblasts is PDGF mediation of microRNA regulation, which may promote differentiation.	(Goff et al., 2008)
mirn140	mirn140 downregulates Pdgf signaling during palatal development. mirn140 function deficiency increases PDGFRA protein levels.	(Eberhart et al., 2008)
mir-103	MiR-103 was increased after baicalintreatment. PDGF-BB-induced abnormal proliferation of smooth muscle cells was inhibited by BA, whereas miR-103 knockdown suppressed proliferation.	(Zhai and Wang, 2022)
miR-26a-5p	In PDGF-BB-stimulated airway smooth muscle cells, the expression of long non-coding RNA LINC-PINT and PTEN was reduced, while miR-26a-5p expression was increased.	(Gao et al., 2021)
miR-128	The signaling pathway of NF-κB can be activated by lncRNA-NEAT1 competitively binding to miR-128, which promotes PDGF-BB-induced inflammatory response and the phenotype transformation of airway smooth muscle cells.	(Song et al., 2022)
miR-1246, miR-182,miR-486	PDGFs regulate exosomal miRNA release from vascular smooth muscle cells (VSMCs). miRNA (miR-1246, miR-182, and miR-486) expression decreased in exosomes derived from PDGF-stimulated VSMCs, which is related with an increase in endothelial cell migration.	(Heo et al., 2020)

**TABLE 4:** Interaction between platelet-derived growth factors (PDGFs) and long non-coding RNA.

Long non-coding RNA	Mutual effects of long non-coding RNA and PDGF	References
lncRNA-NEAT1	The signaling pathway of NF-κB can be activated by lncRNA-NEAT1 competitively binding to miR-128, which promotes PDGF-BB-induced inflammatory response and the phenotype transformation of airway smooth musclecells.	(Song et al., 2022)
lncRNA LINC-PINT lncRNA LINC-PTEN	In PDGF-BB-stimulated airway smooth muscle cells, the expression of long non-coding RNA LINC-PINT and PTEN was reduced, while miR-26a-5p expression was increased.	(Gao et al., 2021)
lncRNA-ANRIL	The phenotypic switchover of vascular smooth muscle cells (VSMCs) to the synthesis of phenotype derived by PDGF was suppressed by the overexpression of lncRNA-ANRIL, which also reversed the decrease in AMPK activity in PDGF-treated VSMCs.	(Hu et al., 2020)
lncRNA LIPCAR	In PDGF-BB- and ox-LDL-treated human VSMCs, the expression of lncRNA LIPCAR was significantly increased.	(Wang et al., 2019)
lncRNA GAS5	In PDGF-BB-treated vascular smooth muscle cells (VSMCs), growth arrest-specific transcript 5 (GAS5) was decreased. PDGF-BB-induced VSMC proliferation and migration was suppressed by overexpression of GAS5.	(Liu et al., 2019)
LncRNA MALAT1	The downregulation of MALAT1 decreased PDGF-BB-induced proliferation and migration by suppressing autophagy.	(Song et al., 2018)
LncRNA HOTTIP	The expression level of long non-coding RNA (lncRNA) HOTTIP was increased in proliferating endothelial cells induced by PDGF-BB.	(Liao et al., 2018)
LncRNA LnRPT	PDGF-BB regulates the expression of lncRNA in pulmonary artery smooth muscle cells.	(Chen et al., 2018b)

A variety of epigenetically related drugs is currently under investigation. For example, DNMT inhibitors can simultaneously upregulate the transcription of neuroprotective genes, including TH genes and PD causative genes, such as *SNCA* and *UCHL1* (Wang et al., 2013). Although studies have confirmed that HDAC inhibitors can exert neuroprotective effects *in vitro* and *in vivo*, their mechanism of action in PD still needs further research (Hegarty et al., 2016). The study of epigenetic modifications in PD is of great significance for discovering potential biomarkers for early diagnosis of PD and its treatment; however, epigenetic modification itself is complex and affected by various factors, such as diet and environment. Therefore, the treatment of PD *via* epigenetic modification targets requires further basic and clinical research.

### Parkinson’s Disease Treatment Based on the Interaction of Exosomes and Platelet-Derived Growth Factors

Exosomes are extracellular vesicles secreted by various kinds of cells. These can be loaded with different cargos, including proteins, lipids, non-coding RNAs, and drugs. Exosomes are ideal carriers as the cargos can be uptaken by target cells without provoking an immune response and avoiding the impediment of the BBB (Wang et al., 2020). Exosomes have been found to play important roles in neurodegenerative diseases, including PD. There is growing evidence that exosomal miRNAs play a key role in PD progression (Cardo et al., 2013; Hoss et al., 2016; Wang et al., 2020). Meanwhile, feasibility of a miRNA exosome-based delivery system has been confirmed in an animal model. Mao et al. found that exosomes carrying miR-34a, released from astrocytes, can enhance the sensitivity of dopaminergic neurons to neurotoxins in a PD model by targeting Bcl-2 (Mao et al., 2015). Moreover, delivery of the loaded drugs to target neuronal cells may increase therapeutic efficacy. For example, Haney et al. developed an exosome-based delivery system for a potent antioxidant, catalase, to treat PD; the catalase-loaded exosomes provided significant neuroprotective effects both in *in vivo* and *in vitro* models of PD (Haney et al., 2015). Yang et al., 2021 demonstrated that exosome-mediated antisense oligonucleotide delivery significantly improved locomotor functions in α-syn A53T mice after exosome–drug injection.

PDGF-loaded exosomes have also been explored. Exosomes, including PDGF-AA derived from differentiating human skeletal myoblasts (HSkM), can induce *in vitro* myogenesis of human adipose-derived stem cells (HASCs) and improve *in vivo* muscle regeneration (Choi et al., 2016). Adipose tissue, bone marrow, and umbilical cord MSCs secrete exosomes holding PDGF-BB, which can promote keratinocyte and dermal fibroblast proliferation and migration (Hoang et al., 2020). The interaction between exosomes and PDGFs is summarized in Table 5.

**TABLE 5:** Interaction between platelet-derived growth factors (PDGFs) and exosomes.

PDGF	Role of exosomes	References
PDGF	Exosomal circ-ATP10A regulates PDGF expression and promotes multiple myelomaangiogenesis.	(Yu et al., 2022)
PDGF	Skin tissues in adipose-derived stem cell exosomes had higher levels of PDGFs than controls.	(Wu et al., 2021)
PDGF	Enrichment of extracellular vesicles (EVs) with activated receptor tyrosine kinases was reduced by inhibiting mTOR signaling in EV donor cells based on PDGF stimulation.	(Gao et al., 2020)
PDGF	PDGF regulates exosomal miRNA release from vascular smooth muscle cells (VSMCs). miRNA (miR-1246, miR-182, and miR-486) expression decreased in exosomes derived from PDGF-stimulated VSMCs, which was related to an increase in endothelial cell migration.	(Heo et al., 2020)
PDGF-A	The change of extracellular matrix composition was simulated by PDGF-A, which is important for the formation of aortic aneurysms through the induction of pathological phenotype switching of SMCs.	(Aschacher et al., 2021)
PDGF-AA	Exosomes including PDGF-AA derived from differentiating human skeletal myoblasts (HSkM) can induce *in vitro* myogenesis of human adipose-derived stem cells (HASCs) and improves *in vivo* muscle regeneration.	(Choi et al., 2016)
PDGF-AA,PDGF-BB	The levels of plasma endothelial cell-derived exosome and platelet-derived exosomeproteins related to atherosclerosis are different in patients with cerebrovascular disease.	(Goetzl et al., 2017)
PDGF-BB	Exosomal miR-301a-3p derived from adipose-derived mesenchymal stem cellscan lighten remodeling and inflammation of airway smooth muscle cells stimulated by PDGF-BB.	(Feng et al., 2022)
PDGF-BB	Adipose tissue mesenchymal stem cells, bone marrow mesenchymal stem cells, and umbilical cord mesenchymal stem cells secreted exosomes containing PDGF-BB, which can promote keratinocyte and dermal fibroblast proliferation and migration.	(Hoang et al., 2020)
PDGF-B	The exosomes released from astrocytes exerted neurotoxic effects on neurons, which was related to increased miR-29b and decreased PDGF-B expression.	(Hu et al., 2012)
PDGFRβ	Activated platelet-derived exosomes including miR-223, miR-339, and miR-21 can be transferred into smooth muscle cells and decrease PDGFR-β expression.	(Tan et al., 2016)

### Parkinson’s Disease Treatment Based on Single-Cell Transcriptomics and Platelet-Derived Growth Factors

As an emerging technology, single-cell transcriptomics supports the direct analysis of gene expression at the single-cell level as well as the analysis of intracellular population heterogeneity and the definition of cell types, cell states, and dynamic transitions of cells. In addition to identifying novel cell subtypes and rare cell populations, single-cell sequencing techniques also contribute to understanding transcriptional dynamics and gene regulatory relationships. For example, Hook et al. (2018) characterized dopaminergic neuronal populations in the mouse brain at embryonic and early postnatal time points using single-cell RNA-seq. Their scRNA-Seq results confirmed 110 marker genes associated with PD in postnatal dopaminergic neurons, demonstrating how this approach opens up a new era in PD gene research (Hook et al., 2018). Lang et al. (2019) mimicked a disease model of PD in iPSC-dopamine neurons and further identified HDAC4 as a regulator of the PD cell phenotype. Their work demonstrates that single-cell transcriptomics can exploit cellular heterogeneity to clarify disease mechanisms and identify therapeutic targets (Lang et al., 2019). In an *in vitro* model of human PD, dopamine neuron-specific stress responses were revealed using single-cell transcriptomics, with implications for cell replacement therapy. The progression of PD involving multiple cell types, such as astrocytes, oligodendrocytes, and microglia, has also been validated with single-cell transcriptomics (Tiklová et al., 2020). Current research on single-cell omics in PD still needs to be developed to improve our understanding of the etiology of PD at the level of different cell types and has an irreplaceable role in targeted therapy.

Single-cell transcriptomics has been used to determine the roles of PDGFs in certain physiological and pathological mechanisms. Based on single-cell transcriptional profiling, Gan et al. found that the PDGF cascade is an important cue in the nucleus pulposus microenvironment of healthy human intervertebral disks (Gan et al., 2021). With the help of single-cell transcriptome profiling of fetal osterix-expressing cells, Bohm et al. identified PDGF-PDGFRβ signaling as a critical functional mediator of skeletal stem and progenitor cell expansion, migration, and angiotropism during bone repair (Böhm et al., 2019). No studies, however, have used single-cell transcriptomics to explore the roles of PDGFs in the pathophysiological mechanisms or treatment of PD.

### Parkinson’s Disease Treatment Based on the Interaction of Clustered Regularly Interspaced Short Palindromic Repeats and Platelet-Derived Growth Factors

The CRISPR-Cas system, a series of RNA-guided endonucleases, is an adaptive immune defense against viruses or foreign gene integration formed by bacteria during long-term evolution. The type II CRISPR system includes four genes (*Cas9*, *Cas1*, *Cas2*, and *Csn1*) and two non-coding RNAs (pre-crRNA and tracrRNA) that can target and degrade foreign DNA in a sequence-specific manner (Garneau et al., 2010). Soldner et al. (2016) used the CRISPR/Cas9 gene-editing method to introduce two mutations into isogenic human pluripotent stem cells, changing only the genes on one chromosome and leaving the other chromosome unchanged. Although one of these two mutations had no effect, the other mutation (from A to G) increased SNCA expression (Soldner et al., 2016). This approach could potentially be used to identify other causative genes in sporadic PD. Wang et al. (2016) also used the CRISPR/Cas9 system to inject Cas9 mRNA and a variety of sgRNAs into Bamaxiang pig prokaryotic embryos, targeting three different loci—DJ-1, Parkin, and Pink1. The piglets appeared healthy and behaved normally, with no manifestations of mutations being identified. This shows that the CRISPR/Cas9 system has great potential in the treatment of PD (Wang et al., 2016). Guhathakurta et al. (2021) further developed a CRISPR/dCas9-based site-specific H3K4me3 demethylation system that recruited the demethylase JARID1A catalytic domain to the SNCA promoter, significantly reducing the level of H3K4me3 modification at the SNCA promoter. At the same time, the level of α-synuclein was significantly reduced (Guhathakurta et al., 2021). The gene-editing technology mediated by the CRISPR system can not only accurately introduce disease-causing genes into specific cells to construct human disease model cells but also achieve repair through homologous recombination mediated by the CRISPR/Cas technology. This provides new opportunities for treatment at the gene level for genetic variant-related neurodegenerative diseases, including PD.

The CRISPR/Cas9 system, as well as PDGFR-B siRNA and pCMV3-PDGFR-B plasmid, was used to identify PDGFR-β as a potential therapeutic target for pterygium (Mai et al., 2019). Lin et al. established a thyroid cancer cell line with 95.6% PDGFRA gene insertion and deletions through CRISPR/Cas9, and demonstrated that xenograft distant lung metastasis was completely eliminated by PDGFRA gene editing; therefore, PDGFRA could be an effective target to inhibit distant metastasis in advanced thyroid carcinoma (Lin et al., 2021). CRISPR/Cas9-mediated depletion of PDGFRβ in cell lines derived from epiretinal membranes of patients with proliferative vitreoretinopathy attenuated vitreous-induced Akt activation and cell proliferation, migration, and contraction (Yang et al., 2020b). Therefore, the CRISPR/Cas9 system is a promising method to better understand the association between PD and PDGFs, and to treat PD with PDGF-derived drugs.

## Current Challenges and Future Directions

As the global population continues to age, the incidence of PD is increasing in a substantial portion of the population. For this reason, the pathogenesis and clinical treatment of PD remain a primary focus of research. Based on our review of the current literature, as a member of the NT family, PDGFs are known to play a significant role in neurodegenerative diseases, and their effects on the course of PD are increasingly considered in the context of CNS cells, including mitochondrial function, oxidative stress, regulation of Ca^2+^ homeostasis, protein folding and aggregation, and neuroinflammation. We have also summarized several possible approaches and opportunities for clinical application.

Although there is still a general lack of research on PDGFs, we believe that the application of PDGFs to the emerging approaches, such as gene precision therapy, stem cell-based therapy, and the exosome-based delivery system, will significantly impact the diagnosis and treatment of PD. As we mentioned before, the study of epigenetic modifications in PD is the key to discovering potential biomarkers for early diagnosis and treatment; since PDGFs have been found to have crucial roles in PD, the potential for PDGF epigenetically related drugs is huge. Moreover, with the help of exosome delivery systems, recipient neuronal cells can be targeted without provoking an immune response and without the impediment of the BBB. In addition, delivery strategies based on NPs can improve the stability of PDGFs and have potential clinical applications. Furthermore, with the advancement of relevant studies, CRISPR/Cas9-mediated alteration of PDGF-related genes in neuronal cells will also be an interesting option to explore.

In the current age of precision gene therapy, it is particularly important to find the right target to treat PD. Single-cell transcriptomics of different neuronal cell types has an irreplaceable role in providing precise targets for this disease. Previous studies have found that PDGFs can not only be epigenetically regulated but also delivered precisely *via* exosomes. However, more cellular and animal studies are needed to prove the roles of PDGFs in these new strategies against PD. The unknown confounding factors, especially in the intricate human microenvironment, present an enormous challenge. As abioscaffold containing PDGFs and other chemokines has been used in the field of bone regeneration (Hao et al., 2021; Ngah et al., 2021), three-dimensional printing, which allows for the creation of suitable biomechanical microenvironments that include PDGFs, may be a valuable strategy to explore in the future.

## Author Contributions

DL, L-TH, QL, and J-HW collected, analyzed, and interpreted current literature, and drafted the manuscript. C-pZ, QL, and J-HW helped revise the manuscript. All of the authors have read and approved the final manuscript.

## Conflict of Interest

The authors declare that the research was conducted in the absence of any commercial or financial relationships that could be construed as a potential conflict of interest.

## Publisher’s Note

All claims expressed in this article are solely those of the authors and do not necessarily represent those of their affiliated organizations, or those of the publisher, the editors and the reviewers. Any product that may be evaluated in this article, or claim that may be made by its manufacturer, is not guaranteed or endorsed by the publisher.
